# Structurally Defined Amphiphilic AAO Membranes Using UV-Assisted Thiol–Yne Chemistry: Applications in Anti-Counterfeiting and Electronics

**DOI:** 10.1021/acsami.4c09040

**Published:** 2024-08-27

**Authors:** Lin-Ruei Lee, Po-Hsin Fan, Yi-Fan Chen, Ming-Hsuan Chang, Yu-Chun Liu, Chun-Chi Chang, Jiun-Tai Chen

**Affiliations:** † Department of Applied Chemistry, National Yang Ming Chiao Tung University, Hsinchu, Taiwan 300093; ‡ Center for Emergent Functional Matter Science, National Yang Ming Chiao Tung University, Hsinchu, Taiwan 300093

**Keywords:** anodic aluminum oxide, click chemistry, thiol−yne reaction, amphiphilic material, anti-counterfeiting, conductive pathways

## Abstract

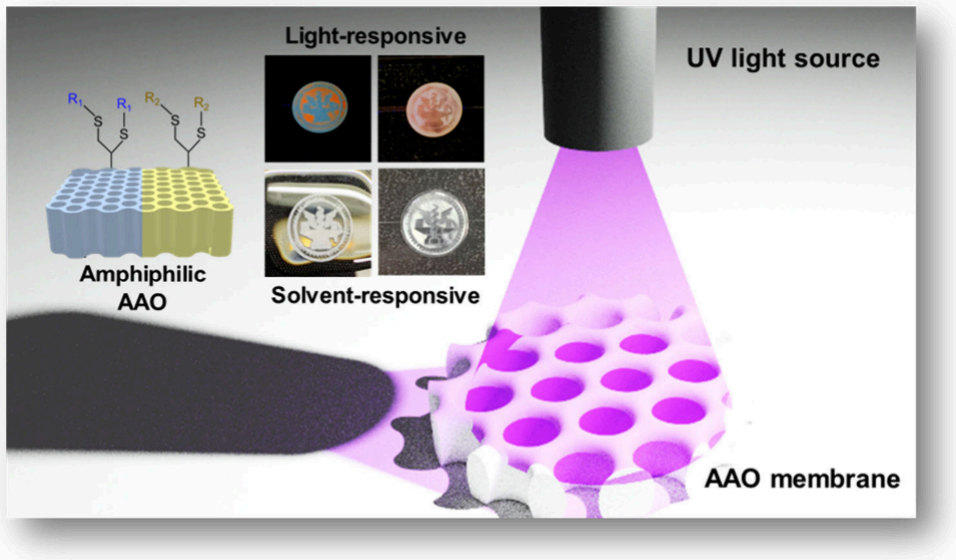

In this study, we fabricate and characterize amphiphilic anodic aluminum oxide (AAO) membranes using UV-triggered thiol–yne click reactions and photomasks for various innovative applications, including driven polymer nanopatterns, anti-counterfeiting, and conductive pathways. Specifically, we synthesize 10-undecynyl-terminated-AAO membranes and subsequently prepare amphiphilic AAO membranes with superhydrophilic and superhydrophobic regions. Various analytical methods, including grazing angle X-ray photoelectron spectroscopy (GIXPS), energy-dispersive X-ray spectroscopy (EDS), scanning electron microscopy (SEM), X-ray diffraction analysis (XRD), nanofocused synchrotron X-ray techniques (nano-XRD and nano-XRF), and water contact angle measurements, confirm the modifications and distinct properties of the modified areas. This work achieves a series of applications, such as driven polymer nanopatterns, solvent- and light-triggered anti-counterfeiting, and region-selective conductive pathways using silver paint with lower resistivity. Besides, the amphiphilic AAO membrane exhibits successful stability, durability, and reusability. To sum up, this study highlights the versatility and potential of amphiphilic AAO membranes in advanced material design and smart applications.

## Introduction

In recent years, the structures containing superhydrophobic (static water contact angles >150°) and superhydrophilic (static water contact angles <10°) patterns have received significant attention and have been developed extensively.^1−3^ Superhydrophobic and superhydrophilic properties both arise from the integration of surface roughness and the nature of surface chemical composition.^2,4−6^ For example, fluoropolymers and butterflies’ wings are well-known superhydrophobic materials because of the intrinsically chemical nature and surface roughness.^3,5,7,8^ In nature, there are also several uniquely superhydrophilic and superhydrophobic structures in living creatures.^5^ For instance, shark skin possesses hydrophilic properties that enable it to reduce the underwater drag, which is essential for a shark’s survival.^5^ In contrast, the lotus leaves exhibit superhydrophobic characteristics, providing them with a highly water-repellent surface.^3,5^ Based on the exclusive properties observed in nature, nature-inspired materials possessing superhydrophilic or superhydrophobic characteristics have attracted attention all along.^2,3,5^ For example, Latthe et al. developed a superhydrophobic coating on several substrates such as a motorcycle body, building walls, a mini boat, a solar panel, window glass, a cotton shirt, fabric shoes, paper currency, plastic, metal, and so on.^1^ Zheng and colleagues also introduced a spray-and-dry method for creating superhydrophilic coatings on glass, polypropylene, and polycarbonate substrates by using colloidal TiO_2_/SiO_2_ nanoparticles.^9^

In nature, there are also some living creatures exhibiting and utilizing both superhydrophobic and superhydrophilic (amphiphilic) features simultaneously for survival.^2,5,10,11^ For instance, the Stenocara beetles in the Namib Desert demonstrate exceptional fog-harvesting abilities because of the hydrophilic–hydrophobic patterned surfaces on their bodies.^2,10−14^ These surfaces enable Stenocara beetles to capture water droplets with hydrophilic patterns and transport them using hydrophobic areas. Therefore, materials that combine both superhydrophobic and superhydrophilic characteristics have gained significant attention and can be extended to promising applications,^14^ including cell growth,^6,15,16^ drug delivery,^17^ fog-harvesting,^13,18−20^ and electronic wetting devices.^21−23^ For example, Wu et al. proposed a reconstructive approach for large-area fabrication of highly hydrophilic patterns on a hydrophobic fluoropolymer coating (RC-HHPS), creating a patterned that serves as an electrowetting platform.^21^

Nowadays, there are several ways to fabricate patterns exhibiting both superhydrophobic and superhydrophilic properties, such as UV light-induced chemical modification,^6,14,24^ stamping,^25^ and photoresist-assisted photolithography.^23,26,27^ Among these methods, patterns with high contrast of wettability fabricated by UV irradiation-assisted chemical modification are more convenient, time-saving, and cost-effective.^6,14,28^ One of the well-known UV irradiation-assisted chemical reactions is thiol–yne click chemistry.^6,14,28,29^ Thiol–yne chemistry involves the reaction between a thiol and a terminal alkyne.^28,29^ Typically catalyzed by a radical initiator, the reaction forms a terminated alkenyl sulfide group through a series of steps with high yields and fast reaction rates.^28,29^ Studies on fabricating patterns with simultaneously superhydrophilic and superhydrophobic properties using thiol–yne click reactions have been widely investigated. Most of these studies, however, focus on fabricating high-contrast wettability patterns for organic polymer films or flat films, and less attention is given to inorganic materials or porous materials. To date, one of well-known porous materials is the anodic aluminum oxide (AAO) membrane. Because of the distinctive features of the AAO membranes, such as high porosity and three-dimensional nano pores, AAO membranes can be utilized in different fields, including drug delivery, filtration, sensors, and growth of nanostructures.^30^ Additionally, anodically oxidized aluminum can be used as the shells of mobile phones.^31^ Therefore, once AAO membranes exhibit amphiphilic properties, their range of applications can be further extended.

To extend the applications of patterns with high contrast of wettability, in this study, we develop amphiphilic porous AAO membranes by integrating the UV-assisted thiol–yne reaction and porous AAO membranes, which possess hexagonal-packed nanopores and high nanopore densities. The amphiphilic AAO membranes are fabricated through a series of chemical modifications using the UV-triggered thiol–yne click reactions with various photomasks. These membranes combine superhydrophilic (hydroxyl-*t*-AAO membrane) and superhydrophobic (fluorine-*t*-AAO membrane) regions within the same membranes, resulting in distinct wettability properties. Through the analyses of grazing angle X-ray photoelectron spectroscopy (GIXPS), energy-dispersive X-ray spectroscopy (EDS), scanning electron microscopy (SEM), and water/solvent contact angles, the amphiphilic AAO membranes are successfully prepared. The unique features of the amphiphilic AAO membranes allow for the precise creation of versatile properties and potential applications such as driven polymer nanopatterns, anti-counterfeiting membranes, and conductive pathways. The fabrication of driven polystyrene (PS) nanopatterns using the amphiphilic AAO membranes, combined with solvents such as toluene, dimethylformamide (DMF), and anisole, demonstrates their potential in patterned nanostructure applications. The results of PS deposition using different solvents are influenced by the viscosities of PS solutions, polymer chain mobilities, and solvent evaporation rates. Additionally, the amphiphilic AAO membranes hold promise for anti-counterfeiting technologies because of their organic solvent-responsive properties. When spiropyran molecules are incorporated into the amphiphilic AAO membranes, forming spiropyran-absorbed AAO (spiropyran-*a*-AAO) membranes, these membranes gain light-responsive properties. The ability to control certain areas’ reactions to organic solvents and UV light enables the development of anti-counterfeiting materials that display specific patterns or changes in response to solvent or light stimuli. The amphiphilic AAO membrane can also create conductive pathways with silver paints. Resistivity measurements by the inductance (L), capacitance (C), and resistance (R) (LCR) meter indicate that the formed conductive pathways using silver paints on the amphiphilic AAO membranes have lower resistivities. This substantial difference emphasizes the potential of these membranes in electronic device fabrication. Besides, the stability, durability, and reusability test are conducted to examine the practicality of the amphiphilic AAO membrane. This study provides a feasible approach to fabricate amphiphilic AAO membranes with versatile features such as distinct wettability, responsiveness to organic solvents and light, and designed conductive pathways, which can be applied in the fields of sensors, anti-counterfeiting membranes, and applied nanomaterials.

## Results and Discussion

**Figure 1:**
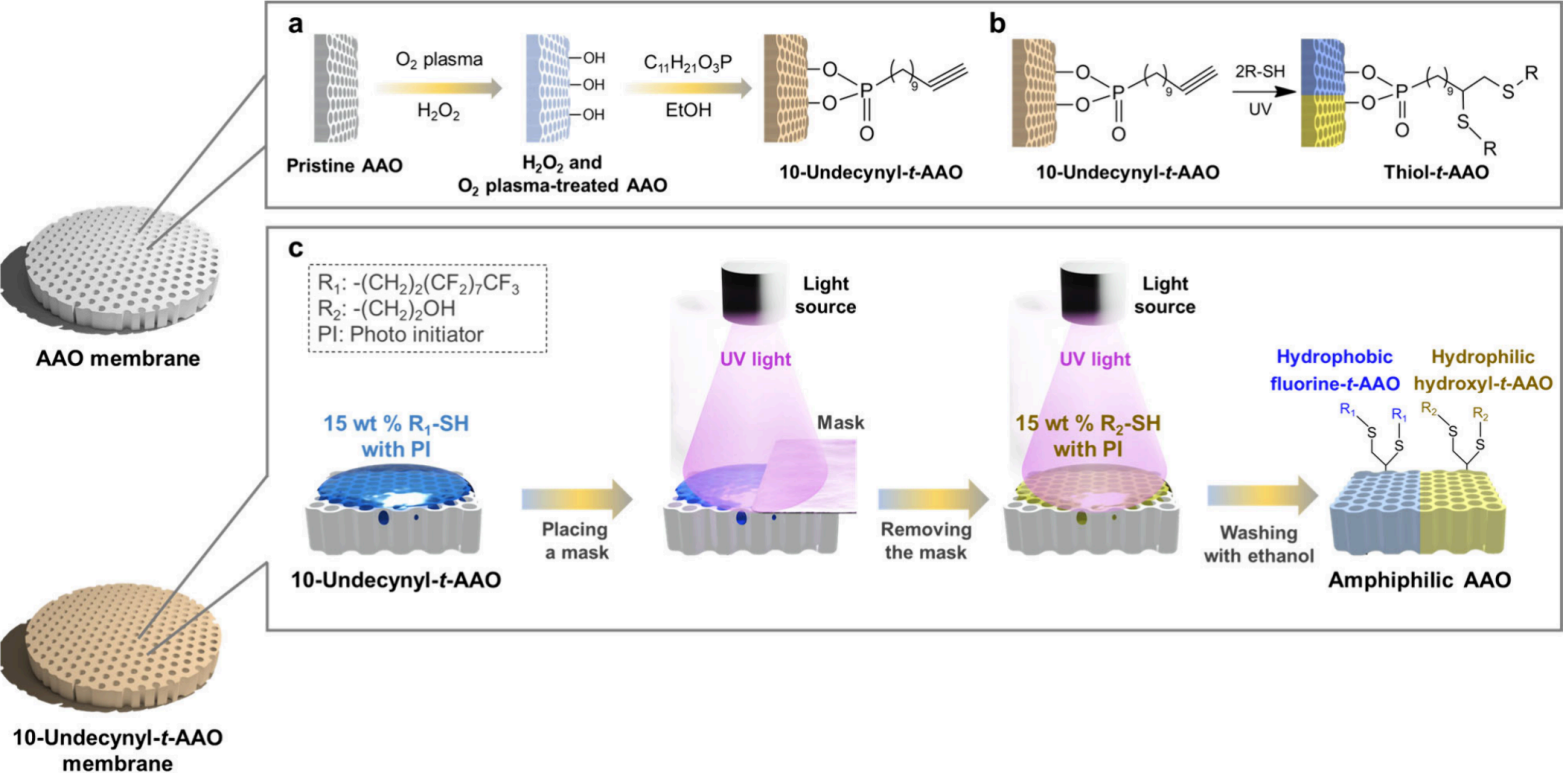
(a) Schematic representation of the preparation of a 10-undecynyl-*t*-AAO membrane. (b) Schematic illustration of UV-triggered thiol–yne click chemistry on an AAO surface. (c) Schematic illustration of fabricating an amphiphilic AAO membrane, which simultaneously exhibits superhydrophilic and superhydrophobic properties on the surface of the AAO membrane.

Figure 1a shows the schematic illustration of the fabrication of a 10-undecynyl-*t*-AAO membrane. An 10-undecynyl-*t*-AAO is produced by inducing OH groups using the H_2_O_2_ solution and O_2_ plasma,^32^ followed by reacting with 10-undecynylphosphonic acid solution to introduce terminal alkyne groups on the AAO surface.^33,34^ In this study, the photoinitiator in the following modification with UV irradiation is used to initiate a radical-mediated thiol–yne click reaction between the thiol and the alkyne groups on the surface of the AAO membrane and to functionalize the AAO surface by producing terminated alkenyl sulfide groups,^6,28,29^ as shown in Figure 1b. The schematic illustration of the fabrication of an amphiphilic AAO is shown in Figure 1c. The amphiphilic AAO membranes, which simultaneously possesses superhydrophilic (hydroxyl-*t*-AAO membrane) and superhydrophobic (fluorine-*t*-AAO membrane) parts on the surface of the AAO membranes, are prepared by a series of UV-trigged thiol–yne reactions with the photomask. In this study, *1H,1H,2H,2H*-perfluorodecanethiol and 2-mercaptoethanol are selected as hydrophobic and hydrophilic reactants, respectively, because of their high reaction rates and ability to induce the largest differences in static water contact angles.^6^ Besides, the reaction time in this study is set as 5 min to ensure that hydrophilic hydroxyl and hydrophobic fluorine functional groups reach saturation on the AAO surfaces. Additionally, the second step of modification does not need a photomask because the alkyne groups fully react with hydrophobic *1H,1H,2H,2H*-perfluorodecanethiol in areas not covered by the photomask.^6^ The real image of the amphiphilic AAO membrane is shown in Figure S1a. It is observed that the surface properties are different with varied wettabilities between the hydroxyl-terminated and the fluorine-terminated parts on the amphiphilic AAO membrane.

**Figure 2:**
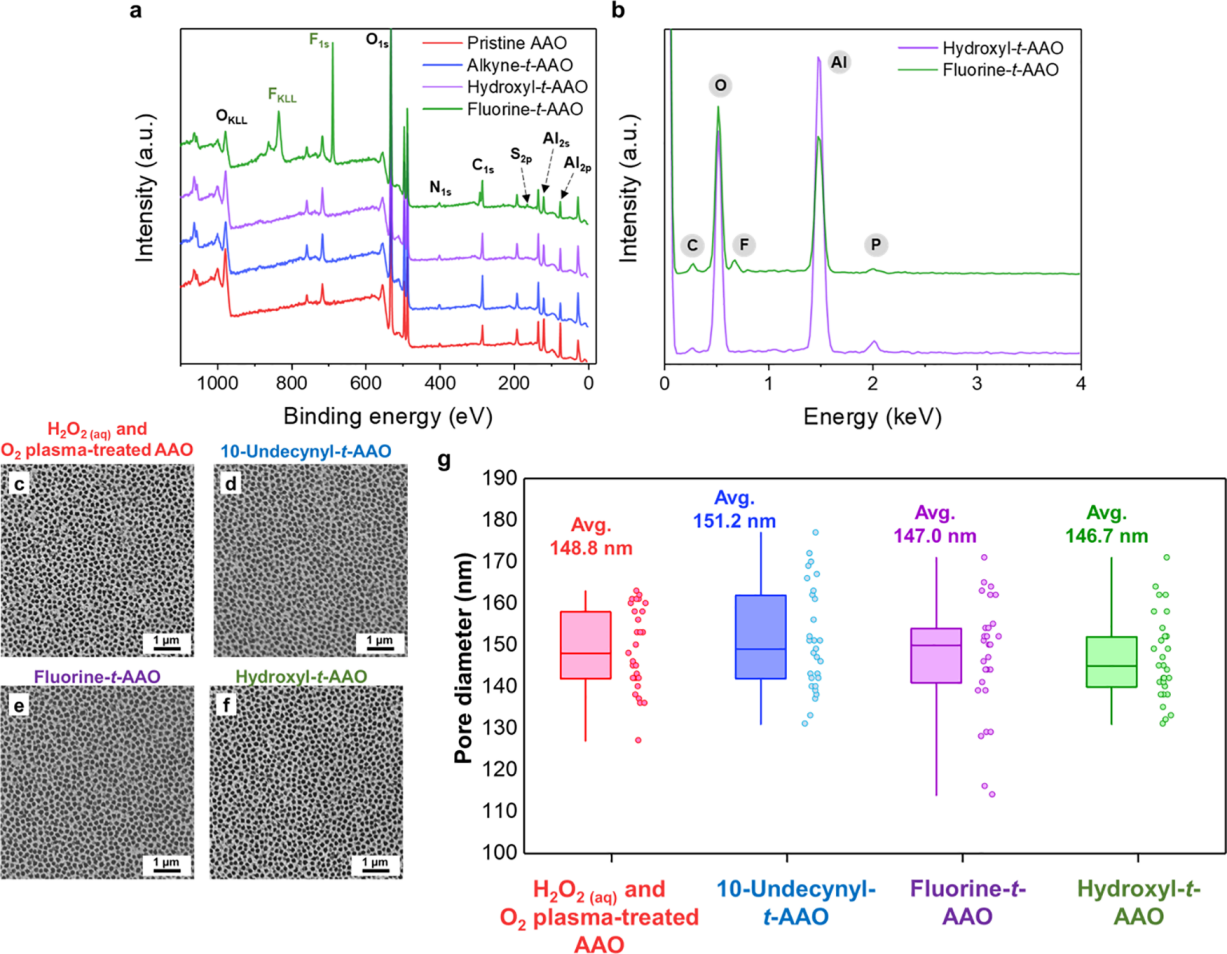
(a) GIXPS spectra of a pristine AAO membrane, an alkyne-*t*-AAO membrane, a hydroxyl-*t*-AAO membrane, and a fluorine-*t*-AAO membrane. (b) EDS spectra of a hydroxyl-*t*-AAO membrane and a fluorine-*t*-AAO membrane. (c–f) SEM images: (c) a H_2_O_2_ and O_2_ plasma-treated AAO membrane, (d) a 10-undecynnyl-*t*-AAO membrane, (e) a fluorine-*t*-AAO membrane, and (f) a hydroxyl-*t*-AAO membrane. (g) Box plots showing pore diameters of AAO membranes before and after modifications.

Angular-resolved XPS and EDS analyses are conducted to provide evidence of the chemical changes between the membranes. XPS spectra with 15° X-ray incidence, as shown in Figure 2a, indicate that there are peaks relevant to S_2p_, N_1s_, and C_1s_ after AAO membranes are chemically modified through UV-triggered thiol–yne click reactions. The result proposes that the terminated fluorine and terminated hydroxyl groups are successfully grafted to the AAO membranes, generating the fluorine-*t*-AAO membrane and hydroxyl-*t*-AAO membrane. As for EDS analysis, as shown in Figure 2b, the K_α_ lines of carbon, fluorine, and phosphorus are observed, indicating that the fluorine-*t*-AAO membrane and hydroxyl-*t*-AAO membrane are formed via thiol–yne chemistry. Except for the chemical information on membranes after surface modifications, the morphological and physical properties are also investigated by using the scanning electron microscope (SEM) and the contact angle meter. From SEM images, revealed in Figure 2c–f, the AAO surfaces demonstrate minimal morphological and roughness variations because the AAO membranes are superficially coated with molecules with low molecular weights.^33,35,36^ Specifically, as shown in Figure 2g, the box plots are used to examine the pore diameters of AAO membranes before and after modifications. The statistical results indicate that the pore diameters remain within the range of 146 to 151 nm after a series of modifications, which demonstrates that the morphologies of the AAO membranes are consistent after the successive modifications. The results of water contact angles of AAO membranes through the chemical modifications shown in Figure S1b, however, indicate that the surface properties change dramatically. After the H_2_O_2_ and O_2_ plasma treatments, the water contact angle of the AAO membrane is ∼10°, suggesting that the presence of hydrophilic surfaces because of the abundance of saturated OH groups. The water contact angle increases to ∼110° after the H_2_O_2_-treated AAO membrane is reacted with 10-undecynylphosphonic acid molecules because of the hydrophobicity of the long carbon chains in the long aliphatic compound.^6,33,36^ For fluorine-*t*-AAO membranes, the water contact angle increases to ∼150° from ∼110° after the 10-undecynyl-*t*-AAO membrane is reacted to *1H,1H,2H,2H*-perfluorodecanethiol with UV irradiation.^6,37^ The result is attributed to the hydrophobicity of functional groups containing fluorine molecules in *1H,1H,2H,2H*-perfluorodecanethiol.^6^ On the other hand, the water contact angle decreases to ∼20° from ∼110° after the 10-undecynyl-*t*-AAO membrane is reacted to 2-mercaptoethanol using the UV irradiation, which is caused by the hydrophilicity of the saturated OH groups on the hydroxyl-*t*-AAO membrane.^6^ Furthermore, the time-resolved static water contact angle measurements are also carried out to investigate the reasons of changes in surface properties. As shown in Figure S2, after 5 min of exposure to 254 nm UV light, the static water contact angles gradually decrease to 20°. Conversely, the static water contact angles steadily increase to 150° with continuous illuminations. The gradual changes in water contact angles on both the hydrophobic and hydrophilic parts once again verify that the hydrophobic properties are attributed to the fluorine groups, while the hydrophilic properties are because of the hydroxyl groups.

**Figure 3:**
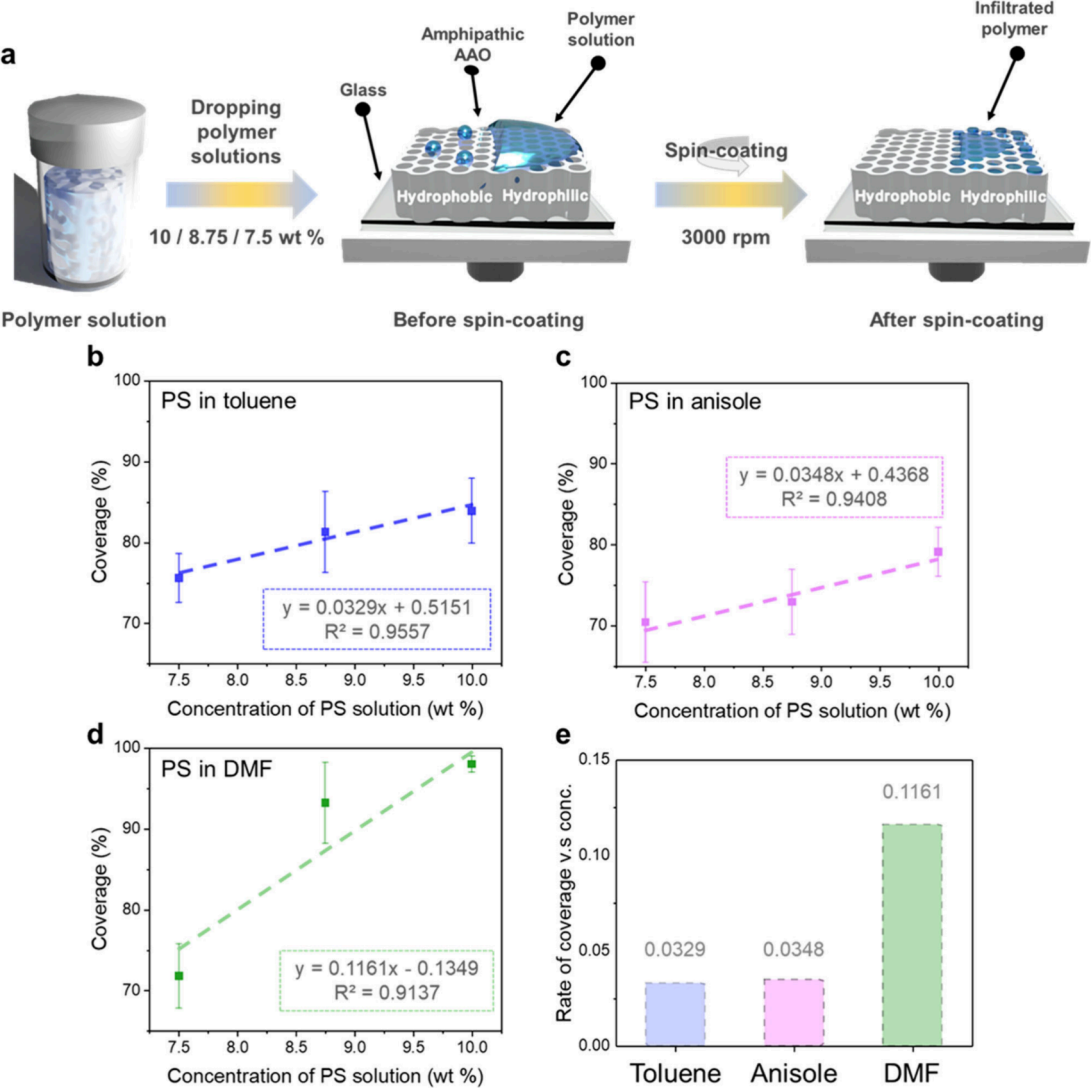
(a) Schematic illustration of the preparation of a driven polymer nanopattern on an amphiphilic AAO membrane. (b–d) Plot of the relation between the coverage and the PS concentrations using (b) toluene, (c) anisole, and (d) DMF as solvents. (e) Plot of the rate of coverages to various concentrations by using different solvents.

To demonstrate the practical applications of the amphiphilic AAO membranes made from the UV-induced thiol–yne click reaction, the amphiphilic AAO membranes are further employed to fabricate driven polymer nanopatterns. Figure S3 depicts the static contact angles on the amphiphilic AAO membrane, including both hydrophobic fluorine-*t*-AAO and hydrophilic hydroxyl-*t*-AAO parts. The result emphasizes that there are larger differences in contact angles between hydrophobic fluorine-*t*-AAO and hydrophilic hydroxyl-*t*-AAO parts using toluene, DMF, and anisole as solvents, which concurs with previous results.^38^ In this study, therefore, toluene, DMF, and anisole are utilized to fabricate the driven polymer nanopatterns on the amphiphilic AAO membranes.^39^ The spin-coating method is used to fabricate the driven polymer nanopatterns on the amphiphilic AAO membranes, as shown in Figure 3a. The driven polymer nanopatterns are prepared by spin coating PS solutions (10, 8.75, and 7.5 wt %) onto the amphiphilic AAO membranes immediately after the PS solutions are applied. The SEM images of driven polymer nanopatterns using toluene, anisole, and DMF as solvents are displayed in Figures S4–S6. In these SEM images, it is emphasized that there are hardly any PSs on the hydrophobic part (fluorine-*t*-AAO membrane), which is attributed to the nature of hydrophobicity combining with centrifugal force.^39,40^ In the hydrophilic part (hydroxyl-*t*-AAO membrane), however, there are different levels of PS coverages on the membranes with various PS concentrations. Therefore, the selective depositions of polymers between the hydrophilic and the hydrophobic regions in the same AAO membrane are achieved.^39^ As shown in the SEM images (Figure S4–S6), it is observed that the PS coverage decreases as the concentration of PS decreases, as presented in the quantitative analysis shown in Figure 3b to 3d. On the other hand, as evidenced by the SEM images, it should be noted that the PS coverages are also influenced by the solvents we use. The coverages of PSs using toluene as solvent are sparse and partially dewetted on the hydroxyl-*t*-AAO membrane, where the aggregated PSs remain. As the concentrations of PSs decrease, there are fewer aggregated areas of dewetted PSs, which transform into infiltrated PSs in the nanopores of the hydroxyl-*t*-AAO membrane. Besides, the infiltrated PSs in the hydroxyl-*t*-AAO membrane caused by capillary force are observed by using anisole as a solvent and the wetted polymers are noticed rather than scattered aggregations. When the concentrations of PSs decrease, it appears that there are decreased amounts of infiltrated PSs. Furthermore, as for the coverage of PSs using DMF as a solvent, the micron-scaled membranous structures of PSs are observed and the nanopores are completely covered by film-like PSs. Once the concentrations of PSs decrease, there are more voids in the PS films on the AAO surface. The behaviors of PS coverings on the hydroxyl-*t*-AAO membrane using toluene, anisole, and DMF as solvents are predominantly caused by the viscosities of the PS solutions, the polymer chain mobilities, and the solvent evaporation rates.^39,41,42^ As shown in Figure 3e, it is recognized that the rate of coverage change versus the PS concentrations is largest with DMF as the solvent. The result is attributed to the film-like structures of PSs, which is not involved in the process of infiltration into the nanopores of the hydroxyl-*t*-AAO membrane. In addition to the concentration effect, the influence of spinning rates on the fabrication of driven polymer nanopatterns is also investigated. Various spinning rates (3000, 4000, and 5000 rpm) are employed to produce nanostructures. As shown in Figures S7–S9, SEM images reveal that changes in spinning rates alter the morphologies of the polymer nanostructures. For the PS-toluene system (Figure S7), an increase in spinning rate leads to the transition of dewetted PS to infiltrated PS, mirroring the effects observed for concentration variations. The transformation is attributed to enhanced polymer chain mobilities and dispersion at higher spinning speeds. Conversely, in the PS-anisole system (Figure S8), increased spinning rates result in the initially infiltrated PS retaining the morphology, with a gradual decrease in coverage because of the elevated spinning speed. In the PS-DMF system (Figure S9), the film-like morphology of PS remains unchanged regardless of the spinning rate. These results indicate that concentration has a more significant impact on polymer dispersions and mobilities than spinning rate does. Moreover, quantitative analyses of PS coverage are conducted. As illustrated in the plots (Figure S10), increasing spinning speeds result in decreased PS coverage in both PS-toluene and PS-anisole systems because of increased dispersion forces and polymer chain mobilities.^39,41,42^ In the PS-DMF system, however, the coverage remains at 100% regardless of the different spinning rates.

**Figure 4:**
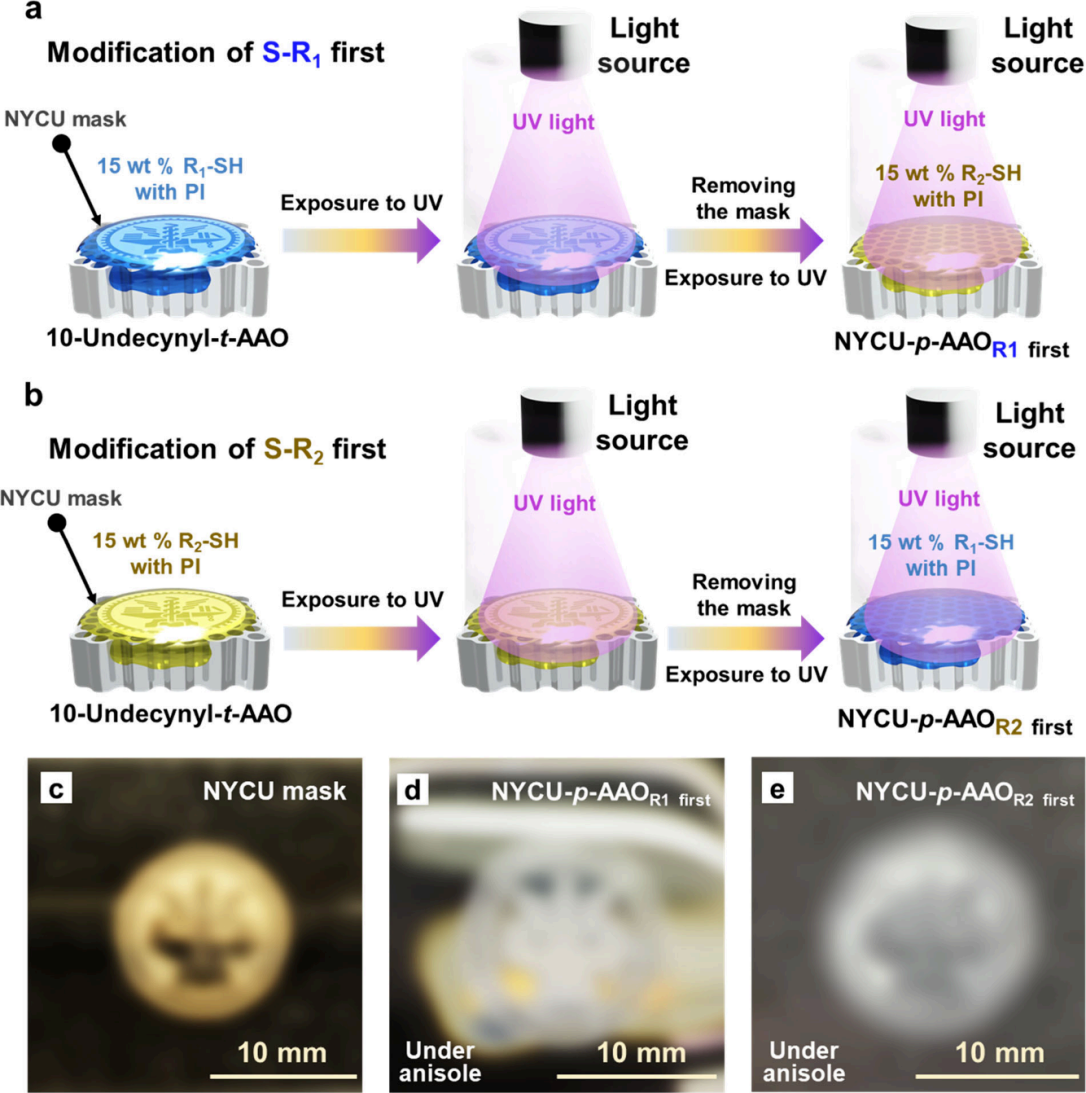
(a) Schematic illustration of the preparation of an NYCU-*p*-AAO membrane by first modifying the S-R_1_. (b) Schematic illustration of the preparation of an NYCU-*p*-AAO membrane by first modifying the S-R_2_. (c–e) Real images: (c) a 12 mm mask with the school emblem of NYCU, (d) an NYCU-*p*-AAO membrane that is initially modified with S-R_1_ and subsequently exposed to anisole, (e) an NYCU-*p*-AAO membrane that is initially modified with S-R_2_ and subsequently exposed to anisole.

The amphiphilic AAO membranes are also applied in the field of anti-counterfeiting.^43^ The anti-counterfeiting AAO membranes are also fabricated by UV-triggered thiol–yne click reaction in conjunction with light-responsive spiropyran.^44^ The anti-counterfeiting AAO membranes are fabricated by two different modification processes. One of them is to modify 10-undecynyl-*t*-AAO membranes with *1H,1H,2H,2H*-perfluorodecanethiol first, as shown in Figure 4a, and the other is to modify 10-undecynyl-*t*-AAO membrane with 2-mercaptoethanol first, as depicted in Figure 4b. The 12 mm mask with a school emblem of NYCU, which is shown in Figure 4c, is first placed on the 10-undecynyl-*t*-AAO membrane, followed by covering with *1H,1H,2H,2H*-perfluorodecanethiol (S-R_1_) or 2-mercaptoethanol (S-R_2_) solutions. The samples are then exposed to 254 nm UV light and washed with ethanol, followed by covered with 2-mercaptoethanol (S-R_2_) or *1H,1H,2H,2H*-perfluorodecanethiol (S-R_1_) solutions and exposed to UV light. By modifying the 10-undecynyl-*t*-AAO membranes with S-R_1_ first or S-R_2_ first, the NYCU-patterned AAO (NYCU-*p*-AAO) membranes are prepared.^6^ Once the organic solvent, anisole, is dropped on the NYCU-*p*-AAO membranes, the school emblem of NYCU is developed on the surface of the AAO membranes, resulting in the solvent-responsive AAO membranes. As shown in Figure 4d, there is a hydrophobic nature in the areas exposed to 254 nm UV light that are not infiltrated with organic solvent (anisole). As seen in Figure 4e, on the other hand, there is a hydrophilic nature in the areas exposed to 254 nm UV light that are not rinsed with organic solvent (anisole). Therefore, the two kinds of solvent-responsive NYCU-*p*-AAO membranes are observed by switching the order of the modifiers, S-R_1_ and S-R_2_. In addition to the solvent-responsive AAO membrane demonstrated by the NYCU-*p*-AAO membrane, the light responsiveness is also emphasized by the NYCU-*p*-AAO membrane after the spiropyran molecule is rinsed on the surface of the NYCU-*p*-AAO membrane.^44,45^

**Figure 5:**
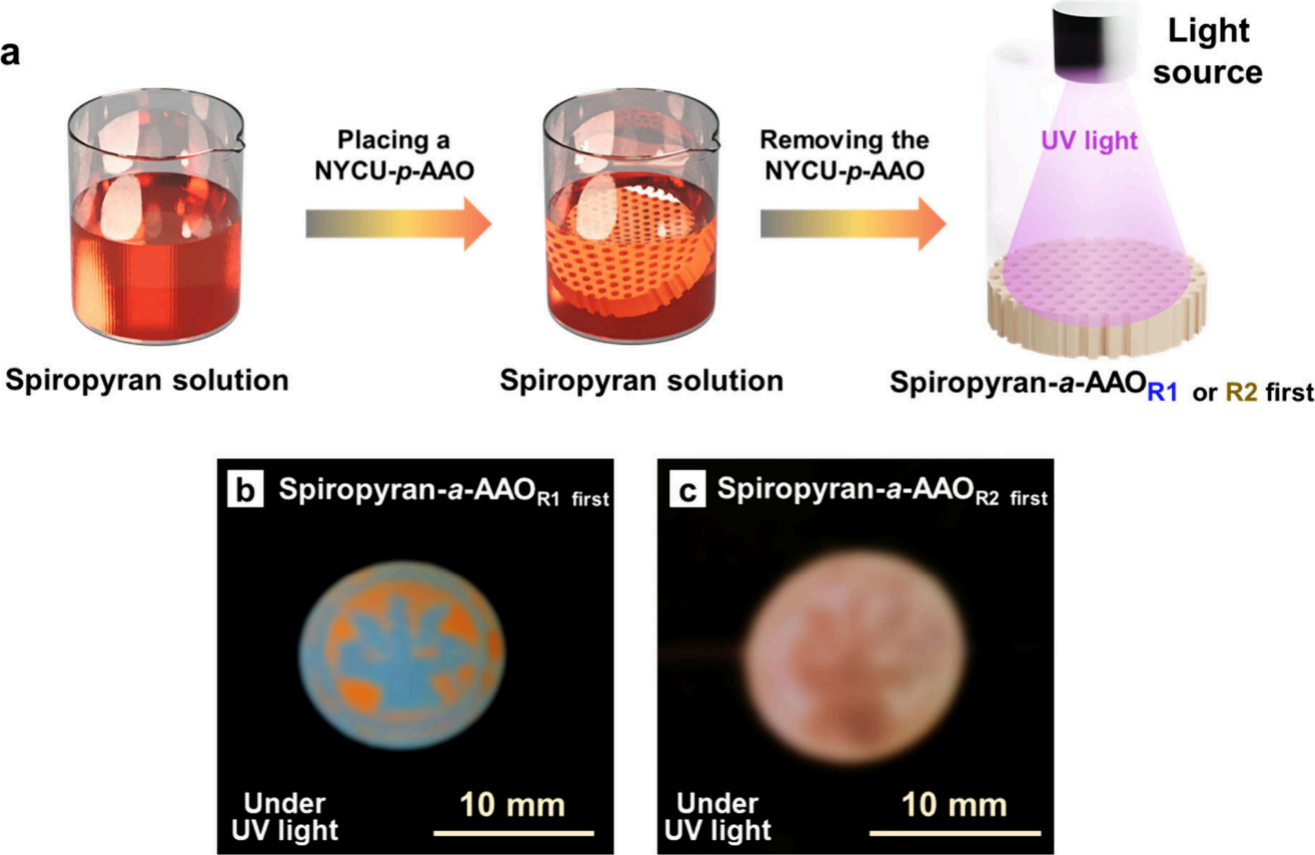
(a) Schematic illustration of the preparation of a spiropyran-*a*-AAO membrane using an NYCU-*p*-AAO membrane. (b, c) Real images: (b) a UV-exposed spiropyran-*a*-AAO membrane, which is created by the NYCU-*p*-AAO membrane that is first modified with S-R_1_, and (c) a UV-exposed spiropyran-*a*-AAO membrane, which is created by the NYCU-*p*-AAO membrane that is first modified with S-R_2_.

**Figure 6:**
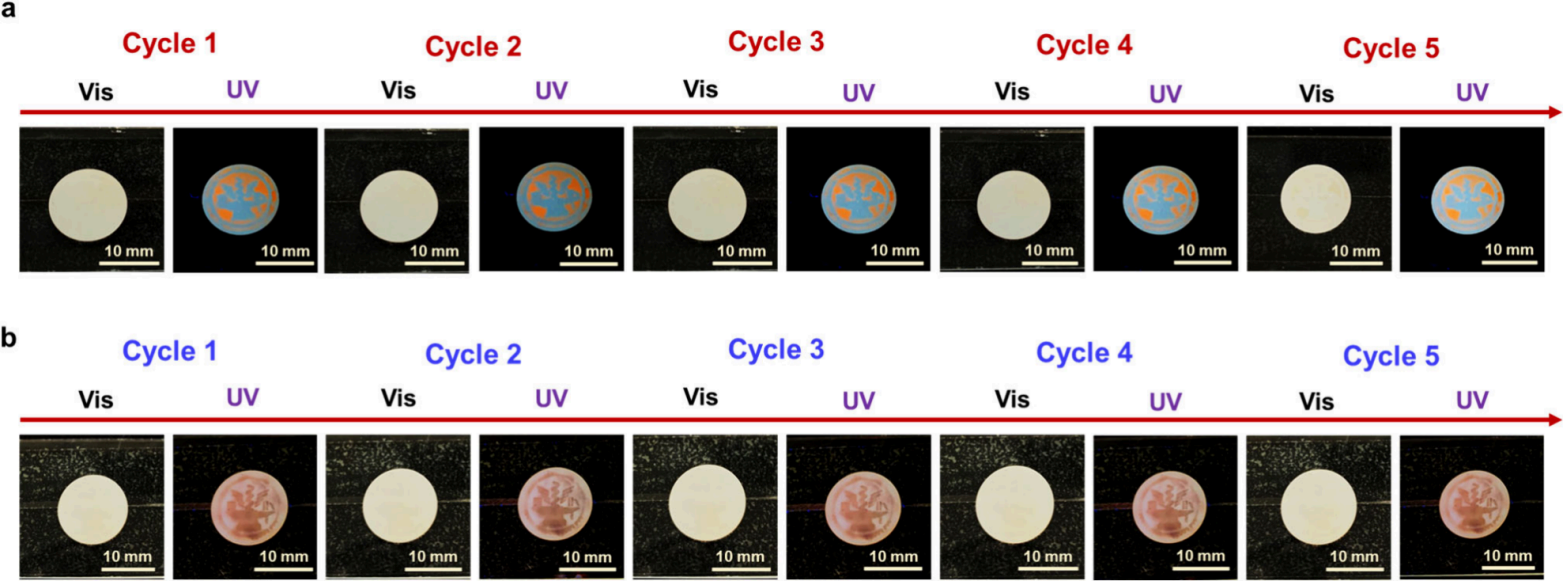
(a, b) Real images of the reversibility test conducted by alternating exposures between UV light and ambient light: (a) the spiropyran-*a*-AAO membrane that is created by the NYCU-*p*-AAO membrane that is first modified with S-R_1_ and (b) the spiropyran-*a*-AAO membrane that is created by the NYCU-*p*-AAO membrane that is first modified with S-R_2_.

Figure 5a shows the schematic illustration of fabricating a spiropyran-absorbed AAO membrane (spiropyran-*a*-AAO membrane). A 0.5 wt % solution of spiropyran in anisole, which has an absorption peak at 365 nm in the UV–vis spectrum (Figure S11a), is first prepared.^45^ Subsequently, the NYCU-*p*-AAO membranes with modification of S-R_1_ or S-R_2_ first are immersed into the solution for 10 s to obtain the spiropyran-*a*-AAO membrane. Figure S11b and S11c show the real images of spiropyran-*a*-AAO membranes that are modified with S-R_1_ first and S-R_2_ first, respectively. The pale white color that is similar to the color of pristine AAO membrane is observed on the spiropyran-*a*-AAO membranes, and there is no such responsiveness without certain stimuli at ambient conditions. When the spiropyran-*a*-AAO membrane is placed under 365 nm UV light, however, the fluorescence with orange color is observed, as presented in Figure 5b and 5c. The result reveals that spiropyran molecule is only absorbed and rinsed in the areas with hydrophilic nature, which are modified with S-R_2_ by 254 nm UV irradiation, glowing the fluorescence under 365 nm UV irradiation.^46^ On the other hand, the areas with hydrophobic nature, which are reacted with S-R_1_ by 254 nm UV light, are not infiltrated by spiropyran molecule. In addition, the reversibility tests of the spiropyran-*a*-AAO membranes are carried out, as shown in Figure 6a and 6b. It is noted that the emitted fluorescence is observed under the test with five consecutive cycles in both spiropyran-*a*-AAO membranes with modification of S-R_1_ or S-R_2_ first. The fluorescent color remains distinct after five cycles, suggesting that the spiropyran-*a*-AAO membranes perform good reversibility.^35^ Therefore, there are two kinds of light-responsive spiropyran-*a*-AAO membranes by switching the order of the modifiers, S-R_1_ and S-R_2_, and further modifying the spiropyran molecules. The light-responsive behavior is similar to that of the NYCU-*p*-AAO membrane, demonstrating its responsiveness to organic solvents.

**Figure 7:**
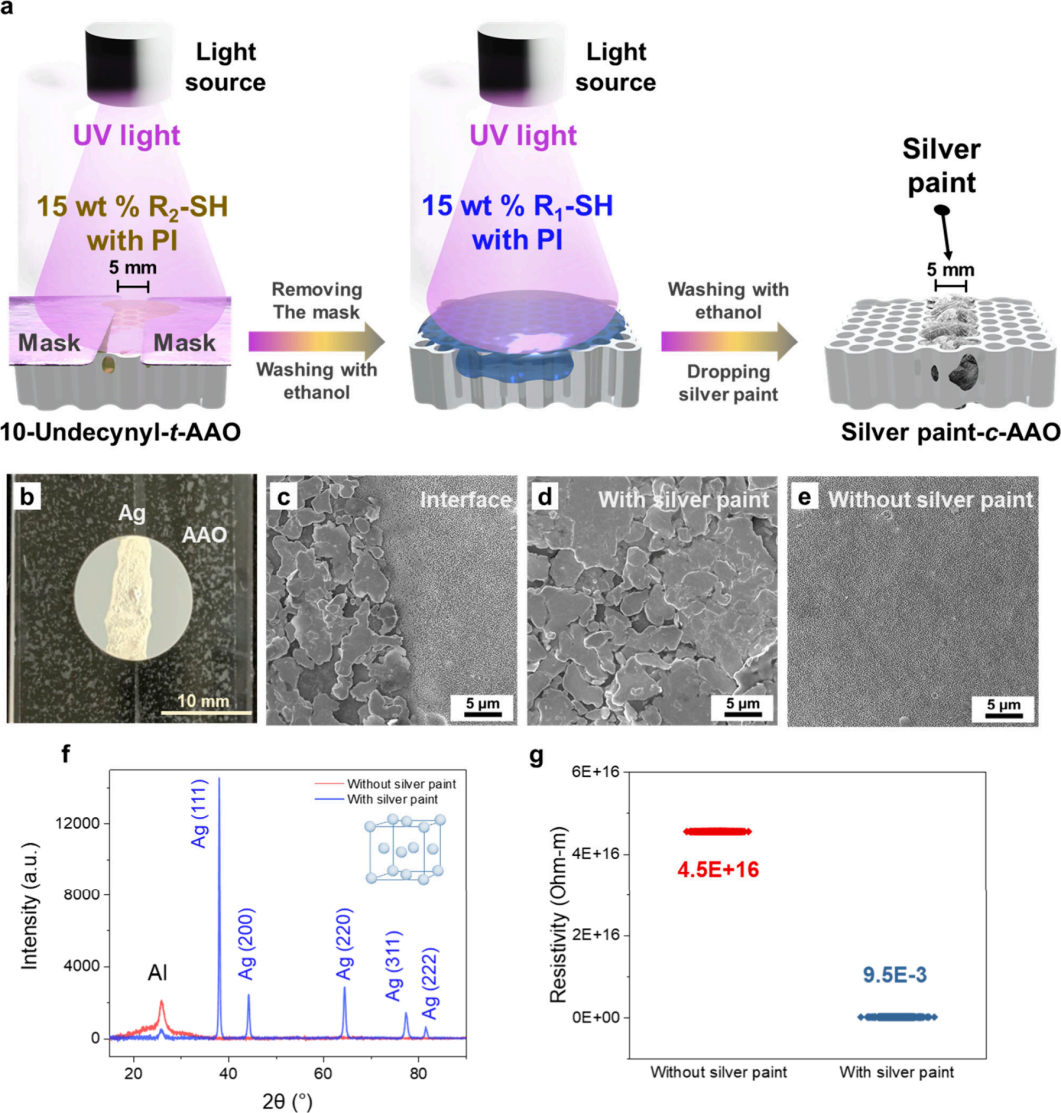
(a) Schematic illustration of the preparation of a silver paint-*c*-AAO membrane. (b) Real image of a silver paint-*c*-AAO membrane. (c-e) SEM images: (c) an interface region of a silver paint-*c*-AAO membrane with and without the silver paints, (d) a hydrophilic region of a silver paint-*c*-AAO membrane that contains the silver paints, and (e) a hydrophobic region of a silver paint-*c*-AAO membrane that does not contain the silver paints. (f) XRD patterns of the regions with and without the silver paints of a silver paint-*c*-AAO membrane. (g) The resistivity tests of the regions with and without silver paints of a silver paint-*c*-AAO membrane.

Aside from demonstrating the responsiveness of amphiphilic AAO membranes to organic solvents and light, the membranes are also used to create conductive pathways.^47^Figure 7a shows the schematic illustration of the preparation of AAO membranes with conductive silver paints coated on certain pathways (silver paint-*c*-AAO membrane). The mask with a 5 mm line width is first placed on the 10-undecynyl-*t*-AAO membrane. After the sample is rinsed with the 15 wt % S-R_2_ solution and exposed to 254 nm UV light for 5 min, the mask is removed, followed by washing with ethanol. The 15 wt % S-R_1_ solution is then dropped on the AAO membrane, followed by exposing to 254 nm UV light again for 5 min. The silver paint is diluted in anisole and dropped on the AAO membrane to form the silver paint-*c*-AAO membrane. As shown in Figure 7b, the diluted silver paint is only deposited on certain pathways that are formed by modifying the AAO membrane with the S-R_2_ solution. The hydrophilic nature caused by the modifier, S-R_2_, induces the silver paint to deposit on certain pathways with a 5 mm line in the middle of the AAO membrane. In the SEM images shown in Figure 7c-e, it is observed that the nodule-like silver paint is only rinsed on certain pathways with the hydrophilic nature and the interface between the areas with and without silver paint is unambiguous. The XRD, EDS, and GIXPS analyses are also utilized to examine the surface properties of the areas with and without the silver paint in the silver paint-*c*-AAO membranes. Figure 7f depicts the XRD patterns of AAO with and without the silver paint. There are peaks representing (111), (200), (220), (311), and (222) lattice planes, which correspond to the lattice planes of silver in the sample of the AAO membrane with silver paint.^48,49^ Besides, the GIXPS spectra shown in Figure S12a reveal that there are peaks corresponding to Ag_3p_, Ag_3d_, Ag_4s_, and Ag_4p_ in the sample of the AAO membrane with the silver paint.^50^ Furthermore, as shown in Figures S12b and S13, the EDS spectra, EDS line scan, and EDS mappings all suggest that the silver paint is located in the areas modified with S-R_2_, which is caused by a hydrophilic nature on the amphiphilic AAO membrane. However, there is no such information about silver paint in the areas modified with S-R_1_, which is induced by a hydrophobic nature in the amphiphilic AAO membrane. In this study, the techniques of nanofocused X-ray fluorescence (nano-XRF) and nanofocused X-ray diffraction (nano-XRD) using a nanosized beam (90 nm^2^) are further conducted to investigate the interface region thoroughly. The beam energy is set at 7 keV, and the scan range is 1000 by 1000 μm^2^ with an interval of 10 μm/step. As shown in Figure S14b,c, nano-XRF mappings reveal prominent signals for aluminum (*K*_α_) and silver (*L*_α_), with noticeable differences in signal intensity between the hydrophilic and hydrophobic regions with distinct boundaries. Furthermore, 2D nano-XRD patterns (Figure S14d,e) show that Ag peaks are exclusively observed in the hydrophilic area, in contrast to the hydrophobic region. Consistent with the nano-XRF results, the nano-XRD mappings (Figure S14f–h) demonstrate significant intensity differences for the Ag (220), (311), and (222) crystal planes between the hydrophilic and hydrophobic regions with clear boundaries. These results, obtained by using the high-resolution synchrotron radiation source, highlight significant differences and distinct boundaries between the hydrophilic and hydrophobic regions. Therefore, the aforementioned results suggest that the deposited silver paint is separated and induced to certain areas on the amphiphilic AAO membranes. The tests for resistivities are also conducted for the amphiphilic AAO membranes with specific conductive silver pathways. To measure the resistivities of the samples, the probes of the LCR [inductance (L), capacitance (C), and resistance (R)] meter are placed on the two terminals that encompass areas with silver paints, forming the conductive pathway as well as areas without silver paints.^51^Figure 7g indicates that the resistivities of the silver paints forming the conductive pathways are ∼9.5 × 10^–3^ Ωm, which is substantially lower than that of the areas without silver paints (∼4.5 × 10^16^ Ωm). Furthermore, to demonstrate the ability to tailor the conductive pathway by using amphiphilic AAO membranes, a photomask with an “N” pattern is used to fabricate the silver paint-*c*-AAO membrane. Initially, the 10-undecynyl-*t*-AAO membrane is modified using S-R_2_ with a photomask featuring an “N” pattern, followed by reacting with S-R_1_. The diluted silver paint is then applied to the AAO membrane, forming the silver paint-*c*-AAO membrane with the “N” pattern. As shown in Figure S15a, the real image of the silver paint-*c*-AAO membrane with the “N” pattern indicates that the silver paint pattern can directly replicate the photomask pattern. Furthermore, AC resistance tests using an LCR meter are carried out to evaluate the conductivity of the conductive pathway with the “N” pattern. As shown in Figure S15b, the results indicate that the resistance of the conductive pathways with the “N” pattern is approximately 4.1 × 10^1^ Ω, which is significantly lower than the resistance of the nonconductive areas (approximately 1.0 × 10^20^ Ω). The results suggest that the conductive pathway can be tailored by altering the patterns of the photomasks. Therefore, the amphiphilic AAO membranes could be further applied in designing conductive pathways using UV-triggered thiol–yne chemistry and specific photomasks.

To extend the practical applications of the amphiphilic AAO membranes, this study also examines their stability, durability, and reusability. Figure S16 demonstrates the stability test of the amphiphilic AAO membrane. The membrane is heated to 100 and 200 °C and then slowly cooled down to room temperature, followed by measuring the static water contact angles of the hydrophobic part. The results indicate that the static water contact angles remain in the range of 144–150°, which demonstrates the good stability of the amphiphilic AAO membrane. Figure S17a shows the durability test of the NYCU-*p*-AAO membrane, which is stored under ambient conditions for 60 days. The distinct pattern of the NYCU emblem remains visible, highlighting the desired durability. Furthermore, Figure S17b presents the reusability test of the NYCU-*p*-AAO membrane. The membrane is alternately immersed in different spiropyran (SP1, SP2, and SP3) solutions, exposed to 365 nm UV light, and then washed with acetone. After exposure to UV light, the membrane exhibits distinct pink, orange, and blue colors in each step, suggesting successful reusability of the amphiphilic AAO membrane. Therefore, all results suggest that the amphiphilic AAO membranes are robust and versatile for various applications.

## Conclusions

In summary, we demonstrate the successful fabrication of amphiphilic AAO membranes through a series of chemical modifications using UV-triggered thiol–yne click reactions with different kinds of photomasks. The amphiphilic AAO membranes integrate distinct wettability properties by combining superhydrophilic (hydroxyl-*t*-AAO membrane) and superhydrophobic (fluorine-*t*-AAO membrane) regions within the same membranes. In this study, the unique characteristic allows for the precise creation of versatile properties and potential applications, such as the driven polymer nanopatterns, anti-counterfeiting membranes, and conductive pathways. The creation of driven PS nanopatterns using the amphiphilic AAO membranes, in conjunction with different solvents such as toluene, DMF, and anisole, demonstrates the potential of these membranes in applications of patterned nanostructures. The unique results of PS depositions using different solvents are based on the viscosities of PS solutions, polymer chain mobilities, and solvent evaporation rates. Besides, the membranes also show promising applications in anti-counterfeiting technologies. By utilizing UV-triggered thiol–yne reactions with spiropyran molecules, the amphiphilic AAO membranes exhibit organic solvent-responsive and light-responsive properties. The ability to control the exposure and reaction of certain areas to organic solvents and UV light results in the development of anti-counterfeiting materials that can display specific patterns or changes in response to solvent or light stimuli. Furthermore, the amphiphilic AAO membrane can create the conductive pathways with silver paints. The resistivity measurements show that the formed conductive pathways using silver paints on the amphiphilic AAO membranes exhibit a low resistivity of 9.5 × 10^–3^ Ωm, which is significantly lower than the resistivity of the areas without silver paints (4.5 × 10^16^ Ωm). This substantial difference highlights the potential of these membranes in electronic device fabrication. Furthermore, the amphiphilic AAO membrane demonstrates the successful stability, durability, and reusability. Overall, this study presents a straightforward strategy for fabricating amphiphilic AAO membranes, which are characteristic of versatile features including their distinct wettability, conductive pathway design, and responsiveness to light and organic solvents. Furthermore, the amphiphilic AAO membranes offer promising applications in sensors, anti-counterfeiting membranes, and applied nanomaterials.

## Experimental Section

### Materials

Hydrogen peroxide (35 wt %) and ethanol (>99.8%) were obtained from Honeywell. Anodic aluminum oxide (AAO) membranes were purchased from Whatman. Acetone (99.5%) and ethanol (99.5%) were bought from Echo. Ethanol (99.9%) was bought Honeywell. Microscope glass slides were bought from DGS. PS (PS, *M*_w_: 26 kg mol^–1^) was obtained from Sigma-Aldrich. 1,3,3-Trimethyl-6-hydroxyspiro (2H-1-benzopyran-2,2-indoline) (99%), *1H,1H,2H,2H*-perfluorodecanethiol (98%), and 2-mercaptoethanol (99%) were purchased from Nova Materials. 2,2-Dimethoxy-2-phenylacetophenone, which is a photoinitiator, was bought from Sigma-Aldrich. Dimethylformamide (99.8%) was bought from Acros. 10-undecynylphosphonic acid (>95%) was bought from SIKÉMIA. Conductive silver paints were purchased from Centenary Materials. The 12 mm mask with a school emblem of NYCU, which was made of stainless steel, was obtained from MicroMetal Technology. 8-Methoxy-1′,3′,3′-trimethyl-6-nitrospiro[chromene-2,2′-indoline] (SP1) and 1′,3′-Dihydro-1′,3′,3′-trimethyl-6-nitrospiro[2*H*-1-benzopyran-2,2′-(2*H*)-indole] (SP2) were bought from UNI-ONWARD Corp. 1,3-Dihydro-1,3,3-trimethylspiro[2*H*-indole-2,3′-[3*H*]naphth[2,1-*b*][1,4]oxazine] (SP3) was purchased from Nova Materials.

### Fabrication of the H_2_O_2_ and O_2_ Plasma-Treated AAO Membranes

An AAO membrane was first placed into a sample bottle containing a 35 wt % hydrogen peroxide solution. Then, the mixture was placed in an oven at 50 °C for 3 h. Subsequently, the AAO membrane was placed into a vacuum chamber and, when the pressure of the chamber was reduced to below 200 mTorr, oxygen gas was introduced. The AAO membrane was exposed to O_2_ plasma with 180 V for 6 min.^32^ After the chamber was returned to ambient pressure, the H_2_O_2_ and O_2_ plasma-treated AAO membrane was fabricated.

### Fabrication of the 10-Undecynyl-*t*-AAO Membranes

A 10-undecynylphosphonic acid solution (2 mg/5 mL) in ethanol was first prepared. The H_2_O_2_ and O_2_ plasma-treated AAO membrane was then placed into the 10-undecynylphosphonic acid solution for 12 h at 50 °C. Subsequently, the AAO membrane was removed from the 10-undecynylphosphonic acid solution. The 10-undecynyl-*t*-AAO membrane was obtained after washed with ethanol and acetone 3 times, respectively, followed by drying under vacuum.

### Fabrication of the Amphiphilic AAO Membranes

The solution of 15 wt % *1H,1H,2H,2H*-perfluorodecanethiol with 0.5 wt % photoinitiator in DMF was first prepared. The solution was then dropped on the 10-undecynyl-*t*-AAO membrane. The photomask, which was made of an aluminum sheet, was then covered on half of the 10-undecynyl-t-AAO membrane, followed by exposure to 254 nm UV light for 5 min. The exposed AAO membrane was then washed with ethanol several times. Subsequently, the solution of 15 wt % 2-mercaptoethanol with 0.5 wt % photoinitiator in DMF was dropped on the half-modified AAO membrane. The amphiphilic AAO membrane was obtained after the AAO membrane was exposed to 254 nm UV light for 5 min, followed by washing with ethanol.

### Fabrication of the Driven Polymer Nanopatterns on the Amphiphilic AAO Membranes

An amphiphilic AAO membrane was placed on the glass substrate. Subsequently, before the spin-coating process start, the tapes were attached on the two edges of AAO membranes to securely fix the AAO membranes on the glass substrates. After the 3 μL PS solution (10, 8.75, or 7.5 wt %) in DMF, anisole, or toluene was dropped on the amphiphilic AAO membranes using a micropipette, respectively, the mixture was spun immediately at 3000 rpm for 60 s. As a result, a driven polymer nanopattern on the amphiphilic AAO membrane was fabricated. On the other hand, as for preparing the driven polymer nanopatterns on the amphiphilic AAO membranes with different spinning rates (3000, 4000, and 5000 rpm), the concentration of PS solutions was kept at 10 wt % using DMF, anisole, or toluene.

### Fabrication of the NYCU-*p*-AAO Membranes with Modification of S-R_1_ First or S-R_2_ First

To fabricate the NYCU-*p*-AAO membrane with modification of S-R_1_ first, a 10-undecynyl-*t*-AAO membrane was covered by a mask with NYCU pattern. After a solution of 15 wt % *1H,1H,2H,2H*-perfluorodecanethiol with 0.5 wt % photoinitiator in DMF was dropped on the mixture, the mixture was exposed to 254 nm UV light for 5 min, followed by washing with ethanol. The mask with NYCU pattern was removed after the first modification. The 15 wt % 2-mercaptoethanol with 0.5 wt % photoinitiator was then dropped on the AAO membrane without the mask, followed by exposing to 254 nm UV light for 5 min. The NYCU-*p*-AAO membrane with modification of S-R_1_ first was obtained after the AAO membrane was washed with ethanol. As for fabricating the NYCU-*p*-AAO membrane with modification of S-R_2_ first, the process was similar to that for fabricating the NYCU-*p*-AAO membrane with modification of S-R_1_ first. The only difference was that, for modification of S-R_2_ first, the 10-undecynyl-*t*-AAO membrane was first modified with 2-mercaptoethanol (S-R_2_), followed by modification with *1H,1H,2H,2H*-perfluorodecanethiol (S-R_1_) without the mask.

### Fabrication of the Light-Responsive Anti-counterfeiting AAO Membranes

A 0.5 wt % solution of 1,3,3-trimethyl-6-hydroxyspiro (2H-1-benzopyran-2,2-indoline) in anisole was first prepared in a sample bottle to make the spiropyran solution. A NYCU-*p*-AAO membrane with modification of S-R_1_ first or S-R_2_ first was then dipped into the spiropyran solution for 5 s. Subsequently, the AAO membrane was removed from the sample bottle. After the AAO membrane was dried with cleaning papers, a light-responsive anti-counterfeiting AAO membrane was obtained.

### Fabrication of the Silver Paint-*c*-AAO Membranes

The 10-undecynyl-*t*-AAO membrane was first covered by a mask with a 5 mm line width. Subsequently, a solution of 15 wt % 2-mercaptoethanol with 0.5 wt % photoinitiator in DMF was dropped on the mixture. The mixture was then exposed to 254 nm UV light for 5 min, followed by washing with ethanol. The mask with a 5 mm line width was removed after the modification. The 15 wt % *1H,1H,2H,2H*-perfluorodecanethiol with 0.5 wt % photoinitiator was then dropped on the AAO membrane without the mask, followed by exposing to 254 nm UV light for 5 min and washing with ethanol. After the silver paint in anisole was dropped on the AAO membrane and the silver residue was removed by cleaning papers, a silver paint-*c*-AAO membrane was obtained.

### Analysis and Characterization

Energy dispersive spectroscope (EDS, Oxford EDS 7585) and grazing incidence X-ray photoelectron spectroscope (GIXPS, ULVAC-PHI PHI QuanteraII) with a grazing angle of 15° were used for surface chemical analysis of the AAO membranes after chemical modifications. The surface morphologies of AAO membranes, AAO membranes after chemical modifications, and AAO membranes with infiltrated PS were examined by a scanning electron microscope (SEM, JEOL JSM- 7401) at an acceleration voltage of 5 kV. Prior to the SEM analysis, the samples were coated with layers of platinum through sputtering. The crystal structures of AAO membranes and those coated with silver paint were investigated using X-ray diffraction analysis (XRD, Bruker D8 Discover X-ray Diffraction System). The crystalline and elemental information around the interface regions in amphiphilic AAO membranes were obtained at the Taiwan Photon Source (TPS) beamline 21A using nanofocused X-ray diffraction analysis (nano-XRD) and nanofocused X-ray fluorescence (nano-XRF) with a synchrotron beam size of 90 nm^2^ at an excitation energy of 7 keV and the scanning area was 1000 by 1000 μm^2^. Water contact angle measurements of AAO membranes and those after chemical modifications were carried out using a contact angle goniometer (FTA125, First Ten Ångstroms) with a CCD camera. The polymer coverage analyses on AAO membranes were conducted by ImageJ software. The resistivity tests of AAO membranes, both uncoated and coated with silver paint, were conducted using an LCR [inductance (L), capacitance (C), and resistance (R)] (GW Instek, LCR-6000) and a total of three hundred data points were collected during the tests.

## Supporting Information Available

Real image of an amphiphilic AAO membrane; images of static water contact angles of a H_2_O_2_ and O_2_ plasma-treated AAO membrane, a 10-undecynnyl-*t*-AAO membrane, a fluorine-*t*-AAO membrane, and a hydroxyl-*t*-AAO membrane; time-resolved static water contact angles of an amphiphilic AAO membrane; static contact angles of a fluorine-*t*-AAO membrane and a hydroxyl-*t*-AAO membrane using different solvents; SEM images of driven polymer nanopatterns on amphiphilic AAO membranes using toluene, anisole, and DMF as solvents with different concentrations and spinning rates; plots of the relation between the polymer coverage and the spinning rate; UV–vis spectra of 1,3,3-trimethyl-6-hydroxyspiro (2*H*-1-benzopyran-2,2-indoline) in anisole before and after exposure to 365 nm UV light; real image of a spiropyran-*a*-AAO membrane; GIXPS spectra of a silver paint-*c*-AAO membrane; EDS spectra, EDS line scan, and EDS mappings of a silver paint-*c*-AAO membrane; nano-XRD and nano-XRF mappings at the boundary between hydrophobic and hydrophilic regions in the silver paint-*c*-AAO membrane; real image of a silver paint-*c*-AAO membrane with an “N” pattern; plot of AC resistance in the regions with and without silver paint of a silver paint-*c*-AAO membrane (PDF)
