# Supplementary material for: Structurally Defined Amphiphilic AAO Membranes Using UV-Assisted Thiol–Yne Chemistry: Applications in Anti-Counterfeiting and Electronics
Source: ACS Appl Mater Interfaces. 2024 Aug 27;16(36):48073–84. doi: 10.1021/acsami.4c09040 (PMC11403548; doi:10.1021/acsami.4c09040)
Supplement: Supplementary file 1 — am4c09040_si_001.pdf [file am4c09040_si_001.pdf]

Supporting Information

Structurally Defined Amphiphilic AAO Membranes Using UV-Assisted Thiol-Yne Chemistry: Applications in Anti-Counterfeiting and Electronics

Lin-Ruei Lee,[1] Po-Hsin Fan,[1] Yi-Fan Chen,[1] Ming-Hsuan Chang,[1] Yu-Chun Liu,[1] Chun-Chi Chang,[1] and Jiun-Tai Chen[*12]

[1.]Department of Applied Chemistry, National Yang Ming Chiao Tung University, Hsinchu, Taiwan 300093

[2.]Center for Emergent Functional Matter Science, National Yang Ming Chiao Tung University, Hsinchu, Taiwan 300093

*email: jtchen@nycu.edu.tw

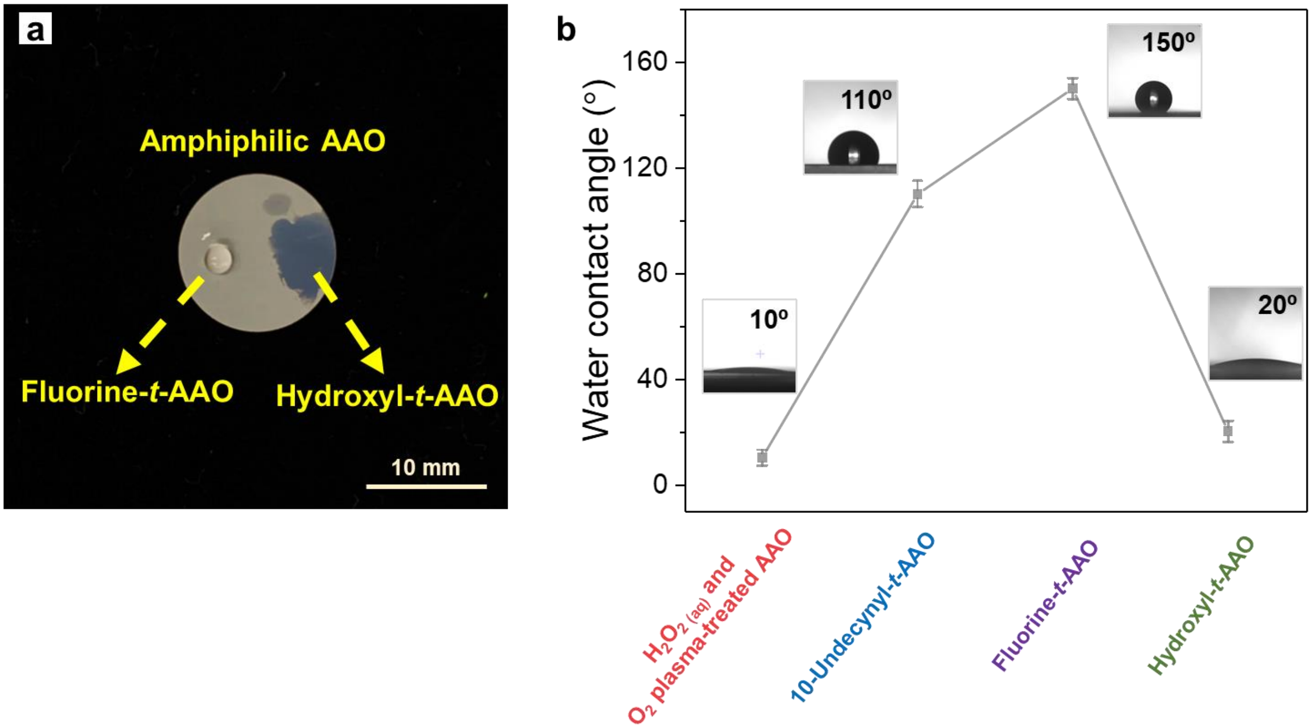

Figure S1. (a) Real image of an amphiphilic AAO membrane. (b) Static water contact angles of an H_2O_2 and O_2 plasma-treated AAO membrane, a 10-undecynnyl-*t*-AAO membrane, a fluorine-*t*-AAO membrane, and a hydroxyl-*t*-AAO membrane.

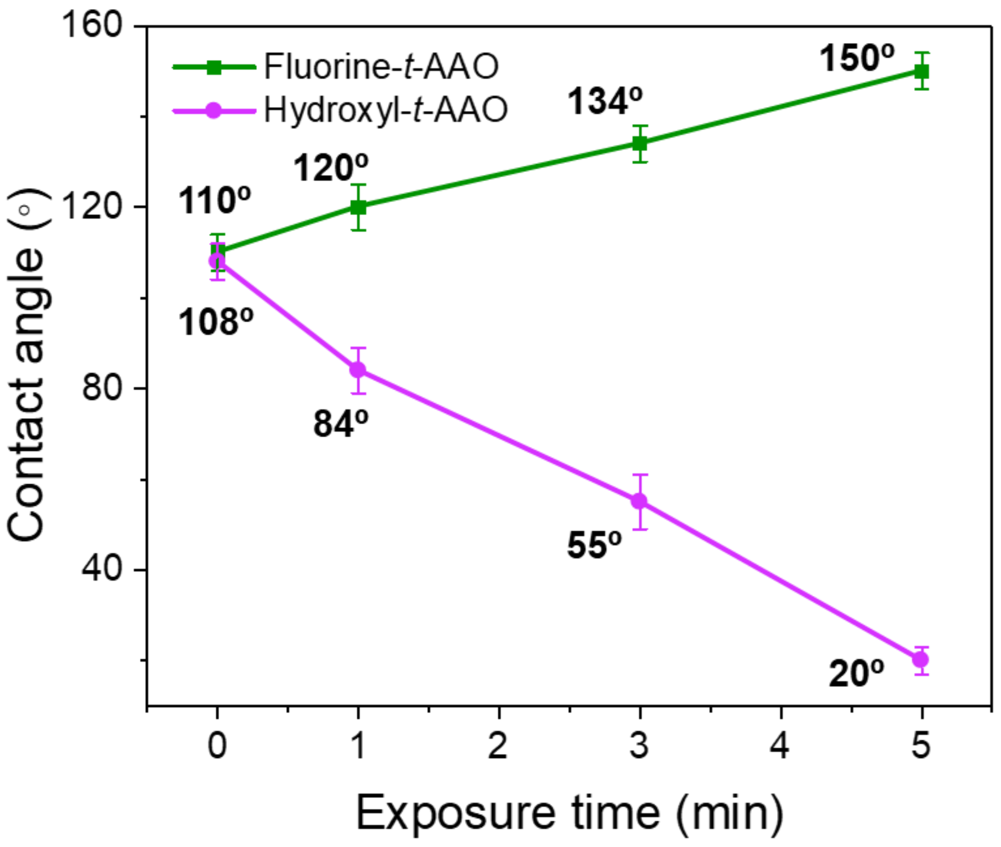

Figure S2. Time-resolved static water contact angles of an amphiphilic AAO membrane. The measurements are carried out on both hydrophobic and hydrophilic parts.

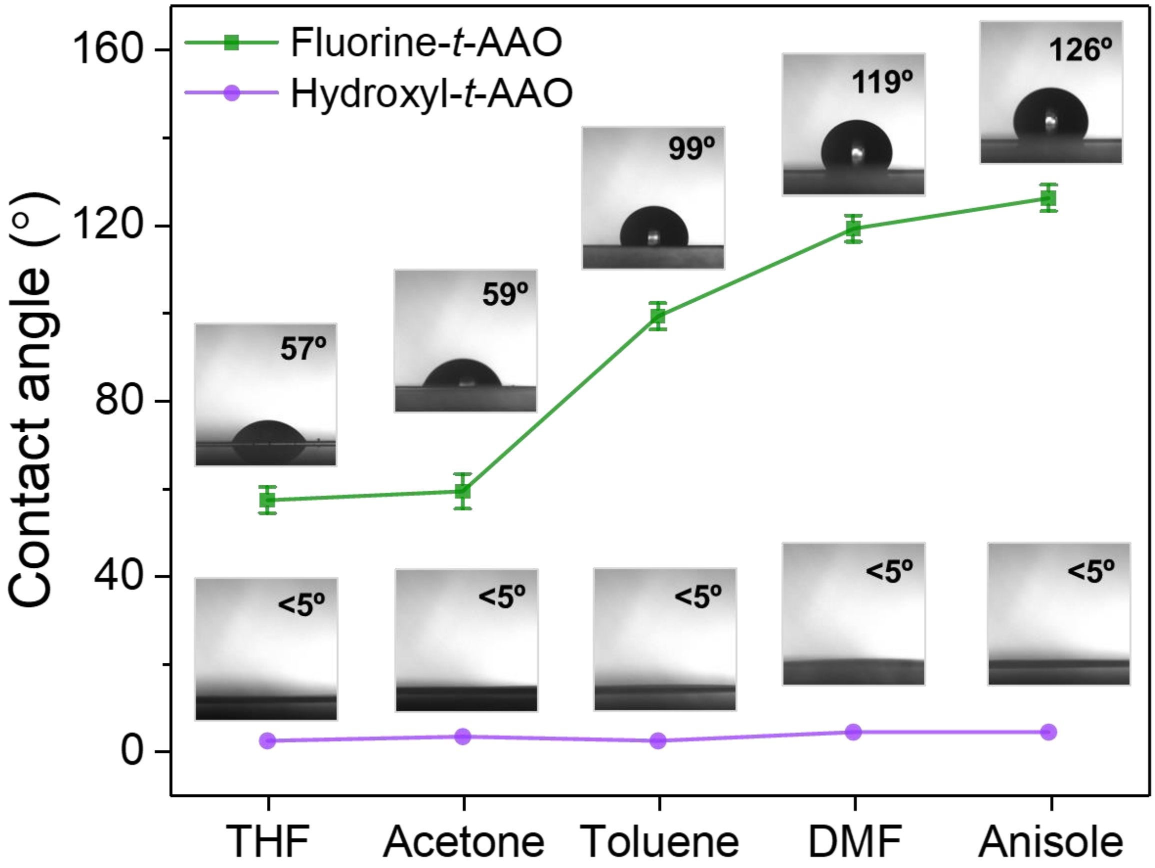

Figure S3. Static contact angles of fluorine-t-AAO membrane and hydroxyl-t-AAO membrane using different solvents: THF, acetone, toluene, DMF, and anisole.

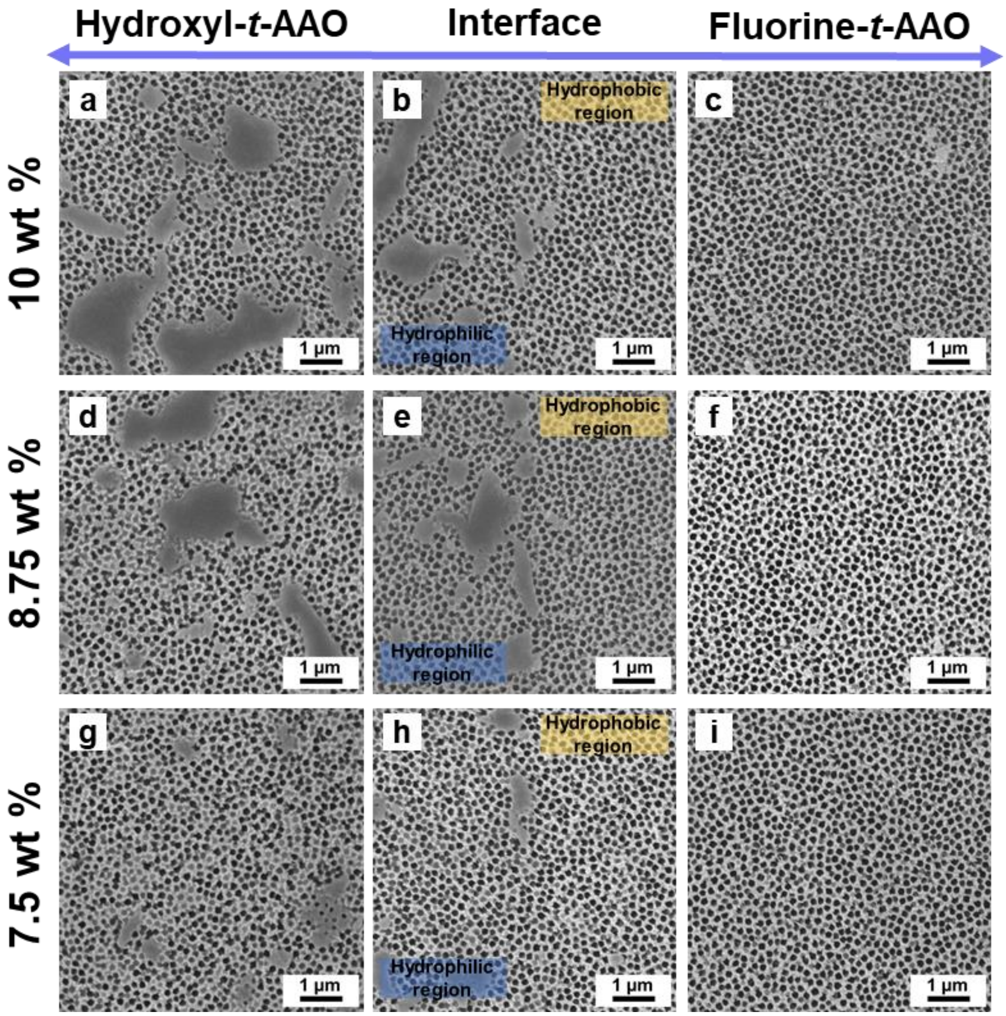

Figure S4. SEM images of driven polymer nanopatterns on amphiphilic AAO membranes in regions of different wettabilities and polymer concentrations: (a) hydrophilic parts using 10, (d) 8.75, and (g) 7.5 wt % of PS solutions; (b) interface parts at 10, (e) 8.75, and (h) 7.5 wt % of PS solutions; and (c) hydrophobic parts at 10, (f) 8.75, and (i) 7.5 wt % of PS solutions. Toluene is used as the solvent to dissolve PS.

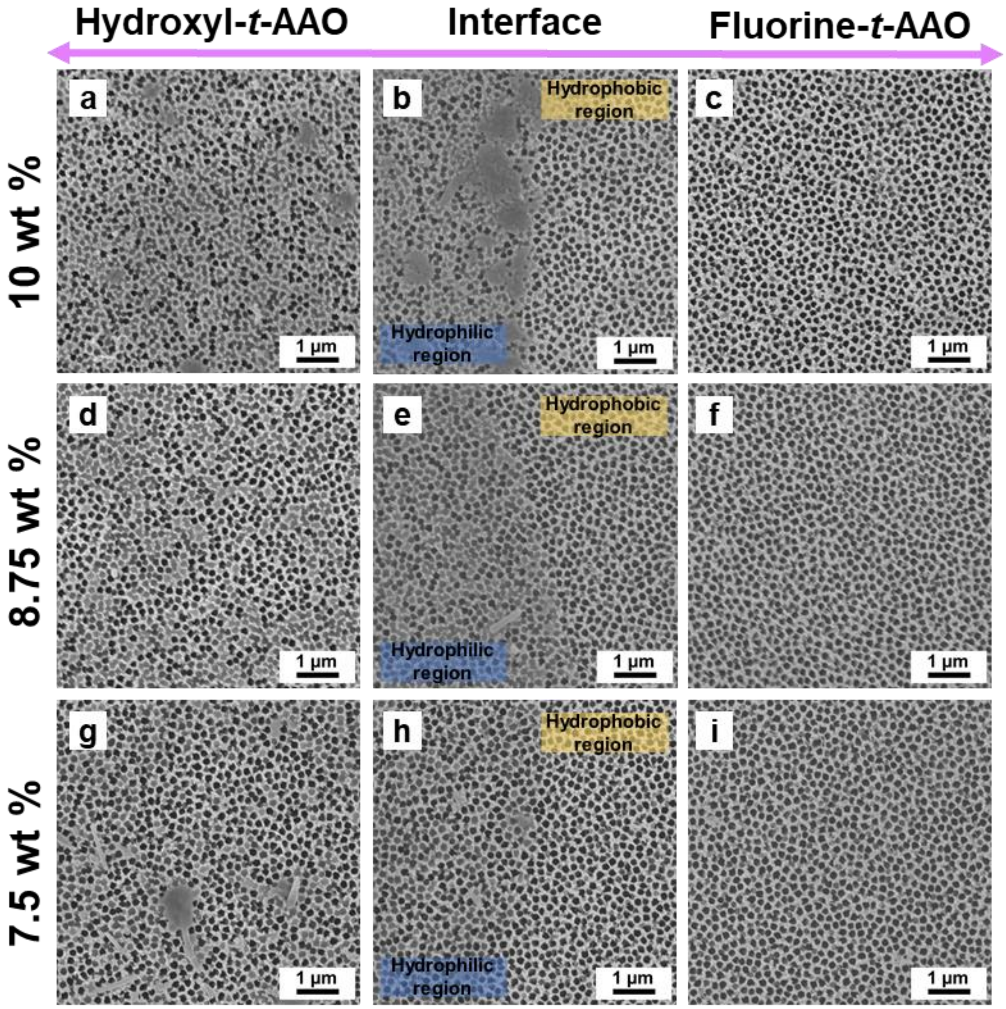

Figure S5. SEM images of driven polymer nanopatterns on amphiphilic AAO membranes in regions of different wettabilities and polymer concentrations: (a) hydrophilic parts using 10, (d) 8.75, and (g) 7.5 wt % of PS solutions; (b) interface parts at 10, (e) 8.75, and (h) 7.5 wt % of PS solutions; and (c) hydrophobic parts at 10, (f) 8.75, and (i) 7.5 wt % of PS solutions. Anisole is used as the solvent to dissolve PS.

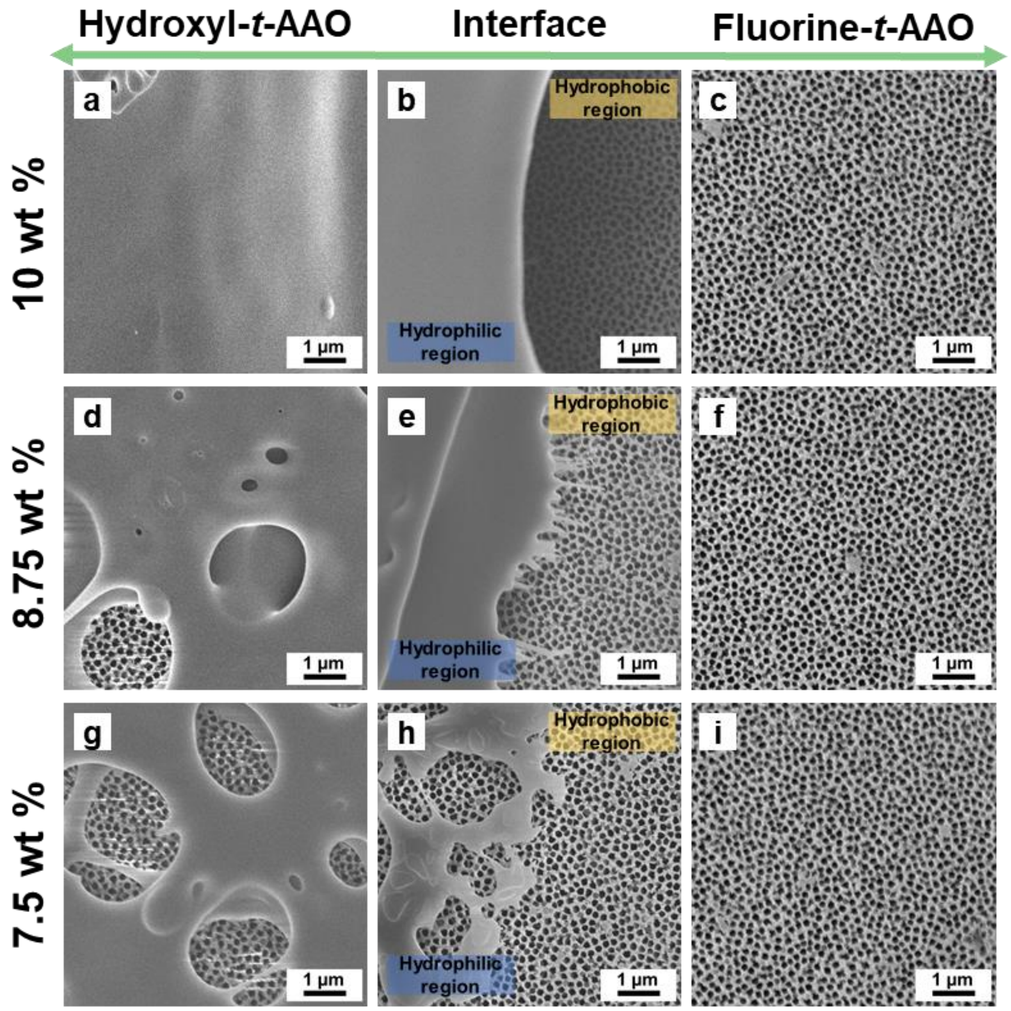

Figure S6. SEM images of driven polymer nanopatterns on amphiphilic AAO membranes in regions of different wettabilities and polymer concentrations: (a) hydrophilic parts using 10, (d) 8.75, and (g) 7.5 wt % of PS solutions; (b) interface parts at 10, (e) 8.75, and (h) 7.5 wt % of PS solutions; and (c) hydrophobic parts at 10, (f) 8.75, and (i) 7.5 wt % of PS solutions. DMF is used as the solvent to dissolve PS.

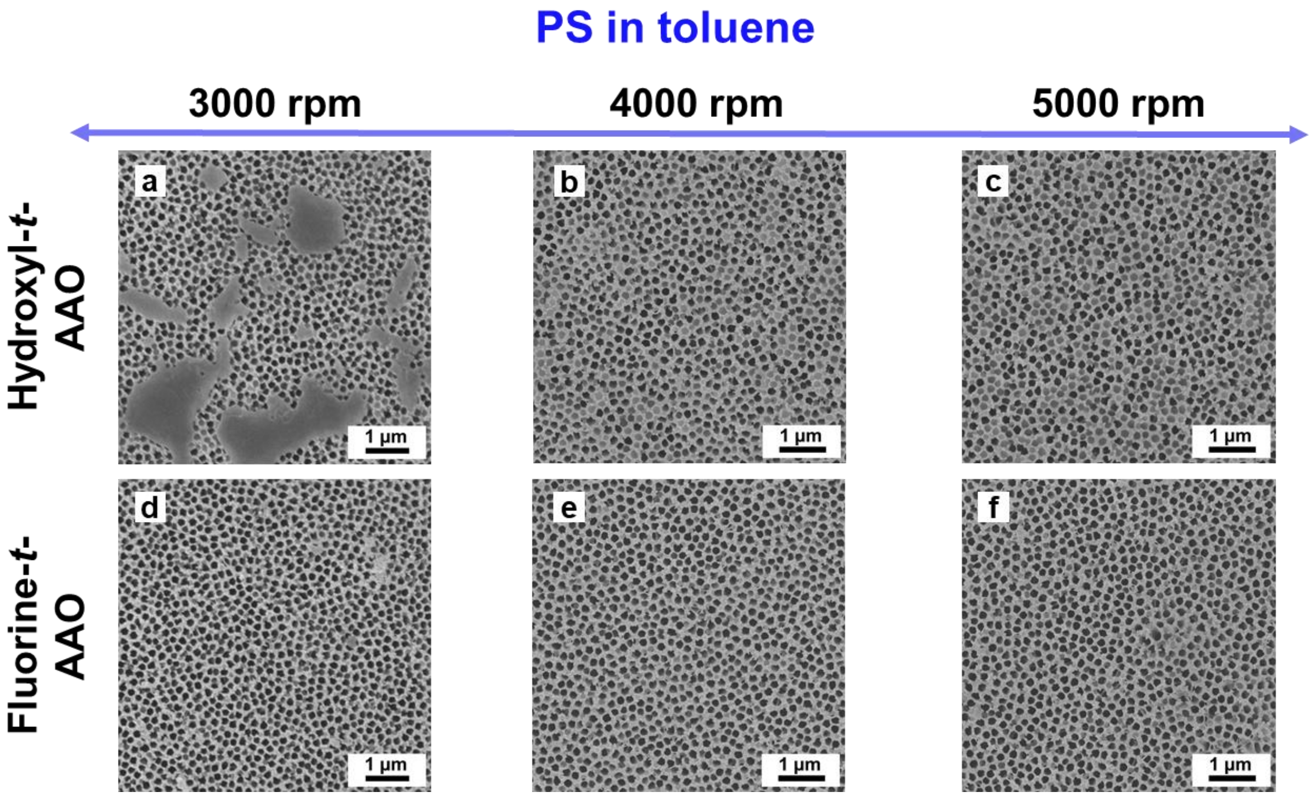

Figure S7. SEM images of driven polymer nanopatterns on amphiphilic AAO membranes in regions of different wettabilities using various spinning rates in spin-coating processes: hydrophilic parts using (a) 3000, (b) 4000, and (c) 5000 rpm; hydrophobic parts using (d) 3000, (e) 4000, and (f) 5000 rpm. Toluene is used as the solvent to prepare the 10 wt % PS solution.

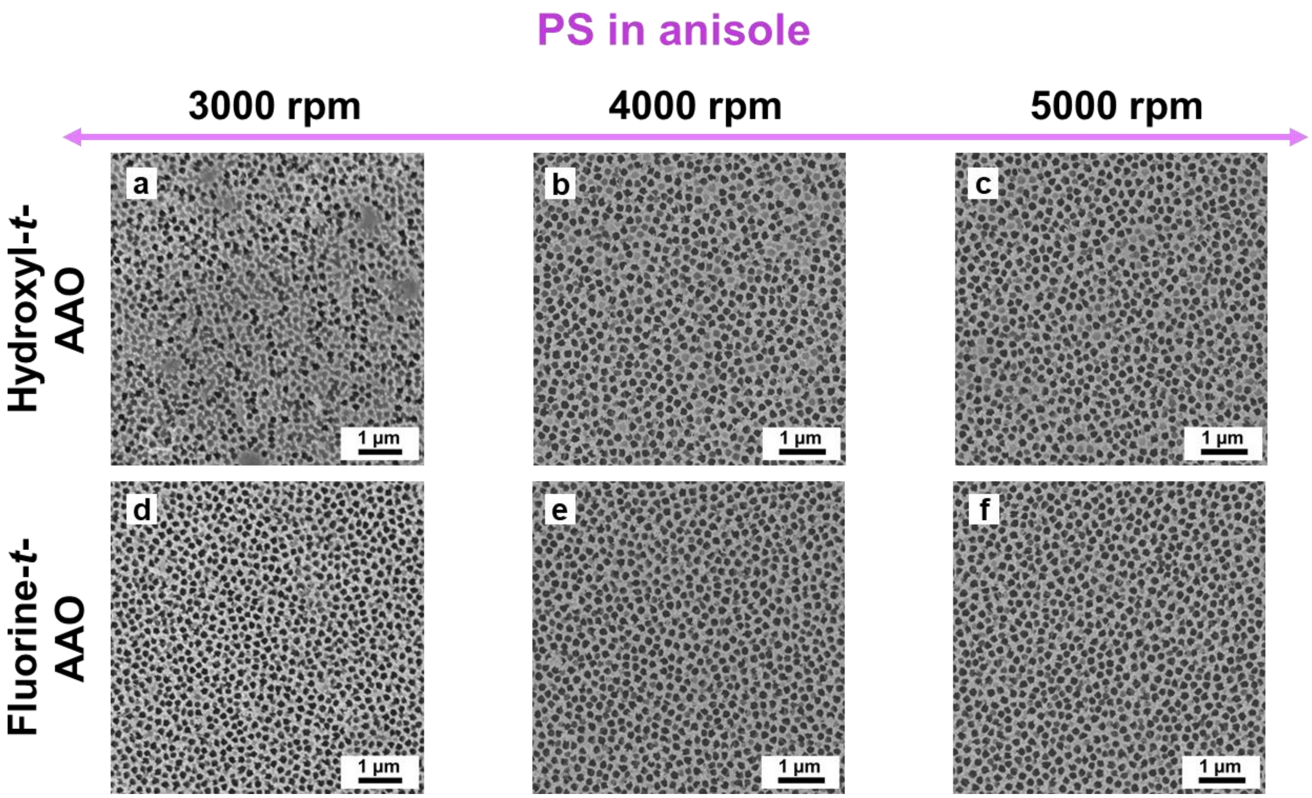

Figure S8. SEM images of driven polymer nanopatterns on amphiphilic AAO membranes in regions of different wettabilities using various spinning rates in spin-coating processes: hydrophilic parts using (a) 3000, (b) 4000, and (c) 5000 rpm; hydrophobic parts using (d) 3000, (e) 4000, and (f) 5000 rpm. Anisole is used as the solvent to prepare the 10 wt % PS solution.

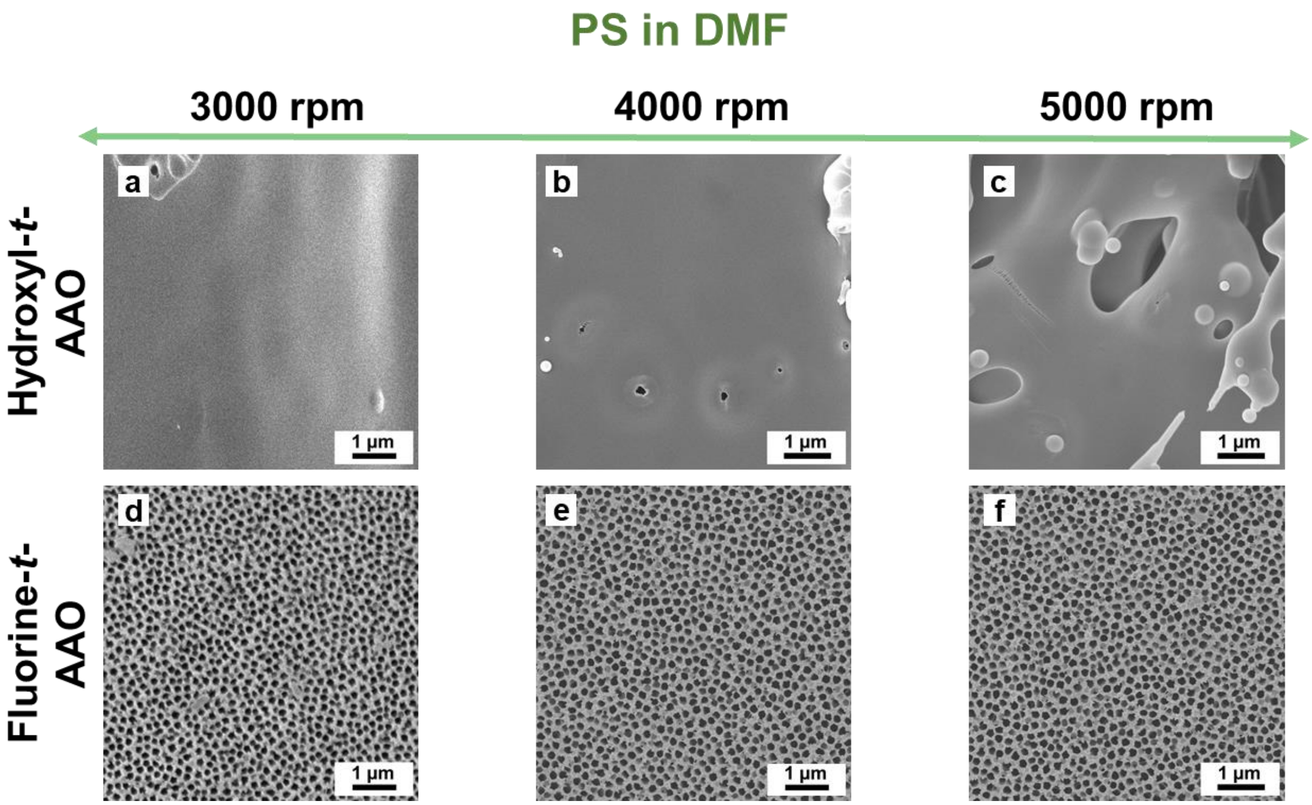

Figure S9. SEM images of driven polymer nanopatterns on amphiphilic AAO membranes in regions of different wettabilities using various spinning rates in spin-coating processes: hydrophilic parts using (a) 3000, (b) 4000, and (c) 5000 rpm; hydrophobic parts using (d) 3000, (e) 4000, and (f) 5000 rpm. DMF is used as the solvent to prepare the 10 wt % PS solution.

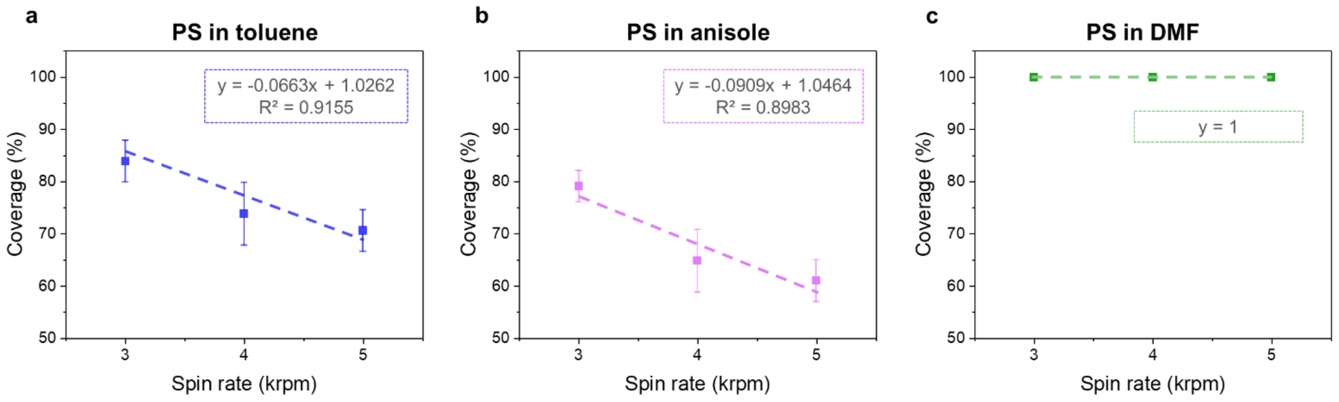

Figure S10. (a-c) Plots of the relation between the polymer coverage and the spinning rate using (a) toluene, (b) anisole, and (c) DMF as the solvents.

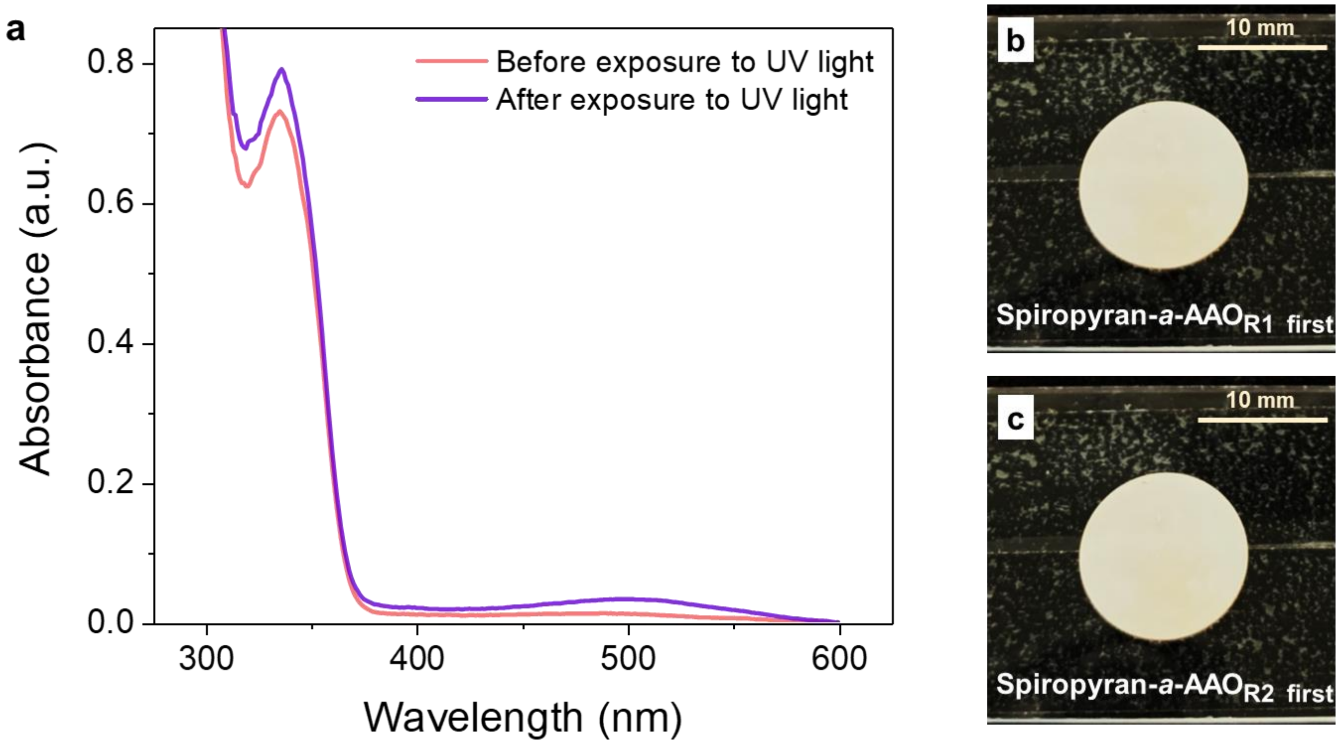

Figure S11. (a) UV-Vis spectra of 1,3,3-trimethyl-6-hydroxyspiro (2H-1-benzopyran-2,2-indoline) before and after exposure to 365 nm UV light. (b-c) Real images of (b) a spiropyran-*a*-AAO membrane that is created by the NYCU-*p*-AAO membrane that is first modified with S-R_1 and (c) a spiropyran-*a*-AAO membrane that is created by the NYCU-*p*-AAO membrane that is first modified with S-R_2.

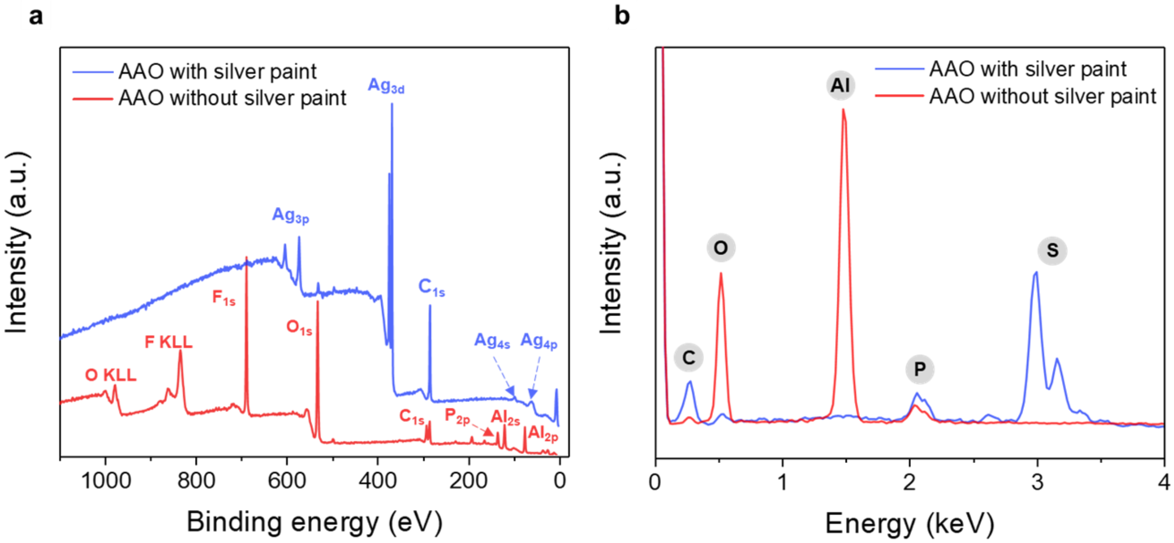

Figure S12. (a) GIXPS spectra of the regions with and without silver paints in a silver paint-*c*-AAO membrane. (b) EDS spectra of the regions with and without silver paints in a silver paint-*c*-AAO membrane.

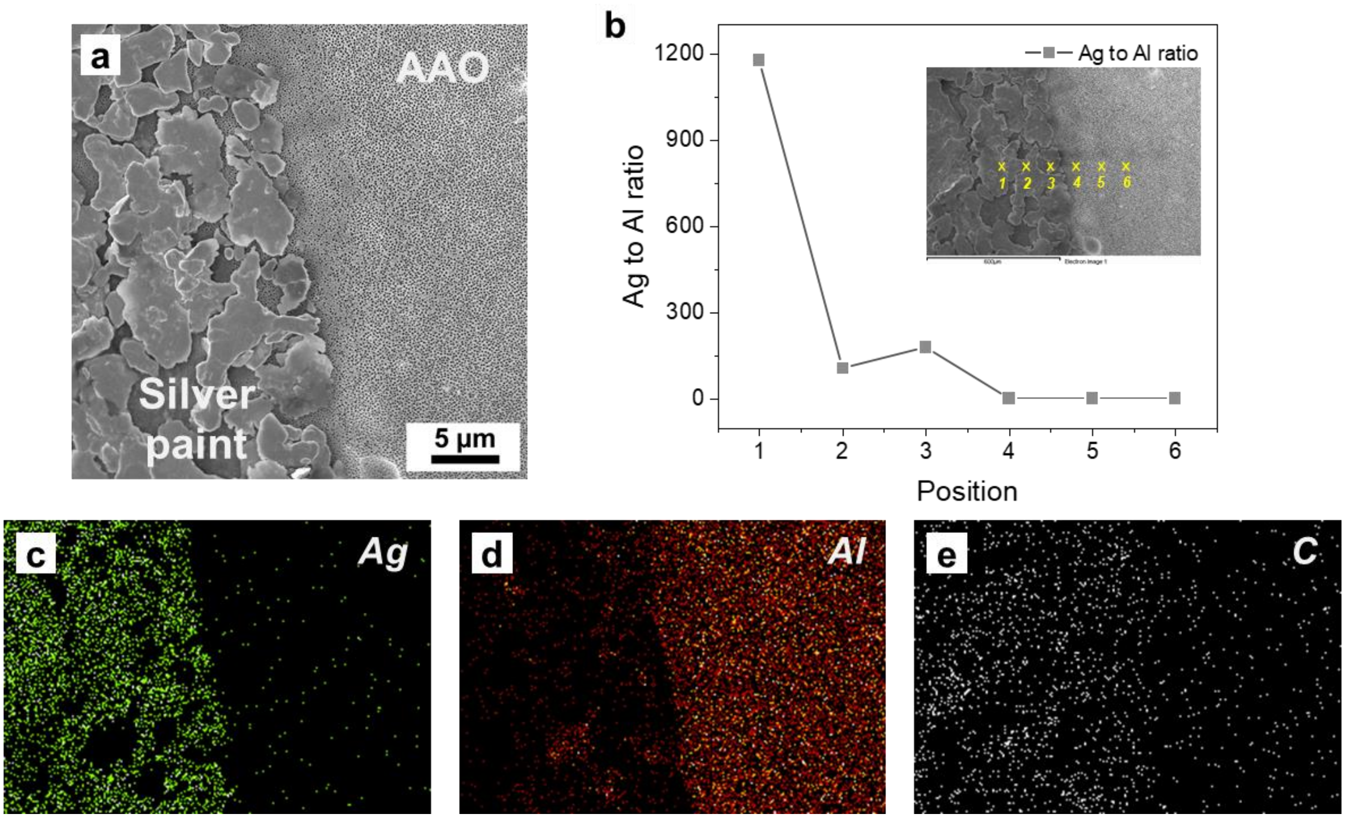

Figure S13. (a) SEM image of the interface region in a silver paint-coated AAO membrane. (b) EDS line scan result showing the Ag to Al ratio at the interface region in the silver-coated AAO membrane. (c-e) EDS mappings of (c) silver (Ag), (d) aluminum (Al), and (e) carbon (C) at the interface region in the silver-coated AAO membrane.

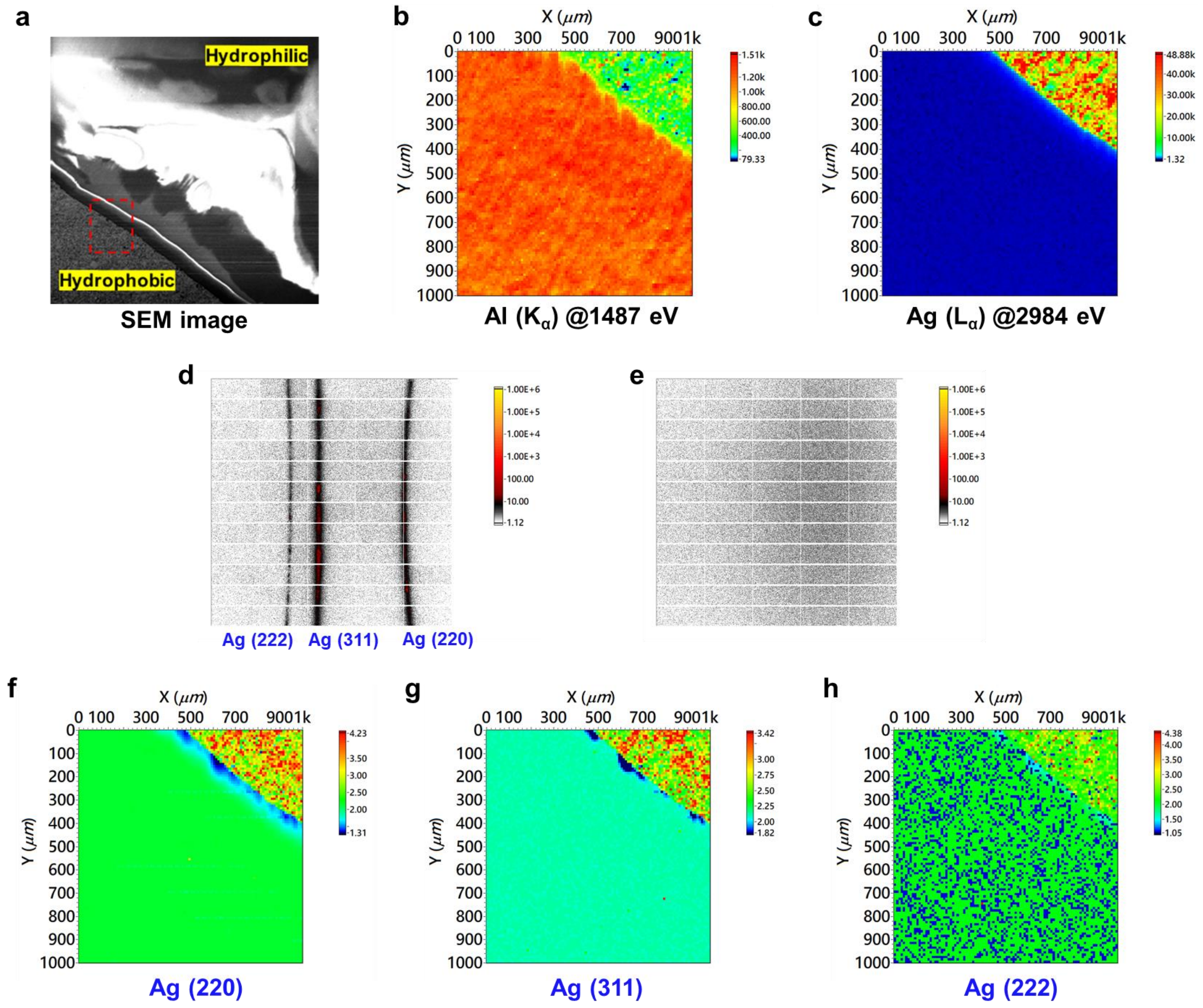

Figure S14. (a) SEM image of the interface region in a silver-coated AAO membrane. The scan area is set at the boundary between hydrophobic and hydrophilic regions. (b-c) Nano-XRF mappings of (b) Al and (c) Ag at the interface region in the silver paint-c-AAO membrane. (d-e) 2D XRD patterns of (d) the hydrophilic region and (e) the hydrophobic region in a silver paint-coated AAO membrane. (f-g) Nano-XRD mappings of (f) Ag (220), (g) Ag (311), and (h) Ag (222) crystalline planes at the interface region in a silver-c-AAO membrane.

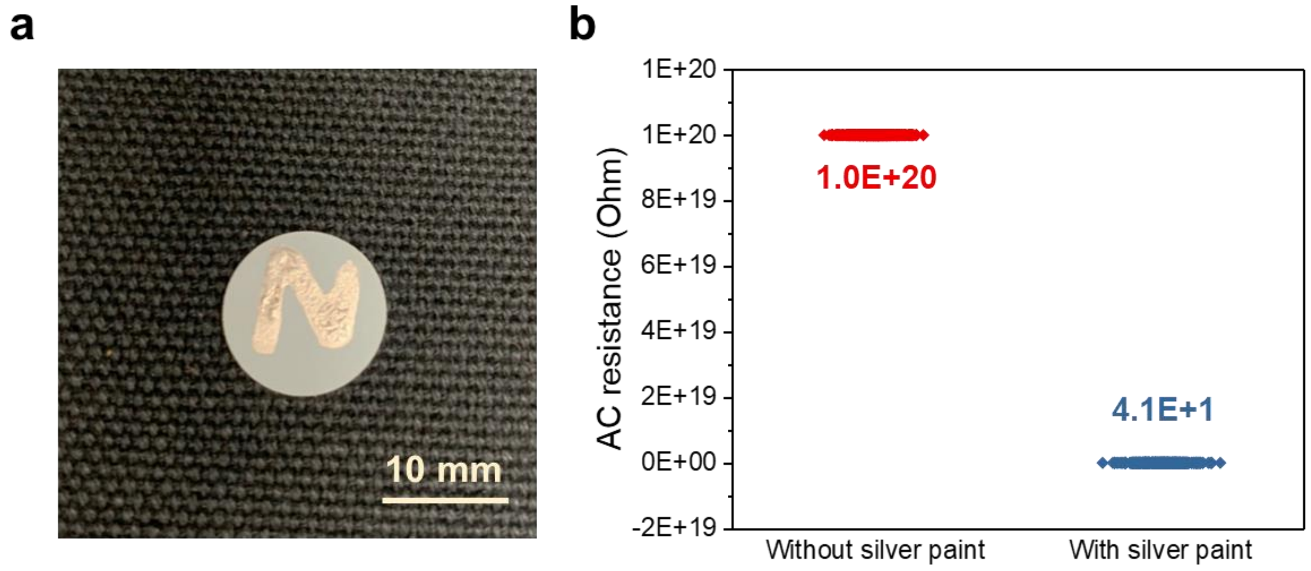

Figure S15. (a) Real image of a silver paint-*c*-AAO membrane with an "N" pattern. (b) Plot of AC resistance in the regions with and without silver paints of a silver paint-*c*-AAO membrane. In this experiment, a photomask with the letter "N" pattern is used to fabricate the silver paint-*c*-AAO membrane.

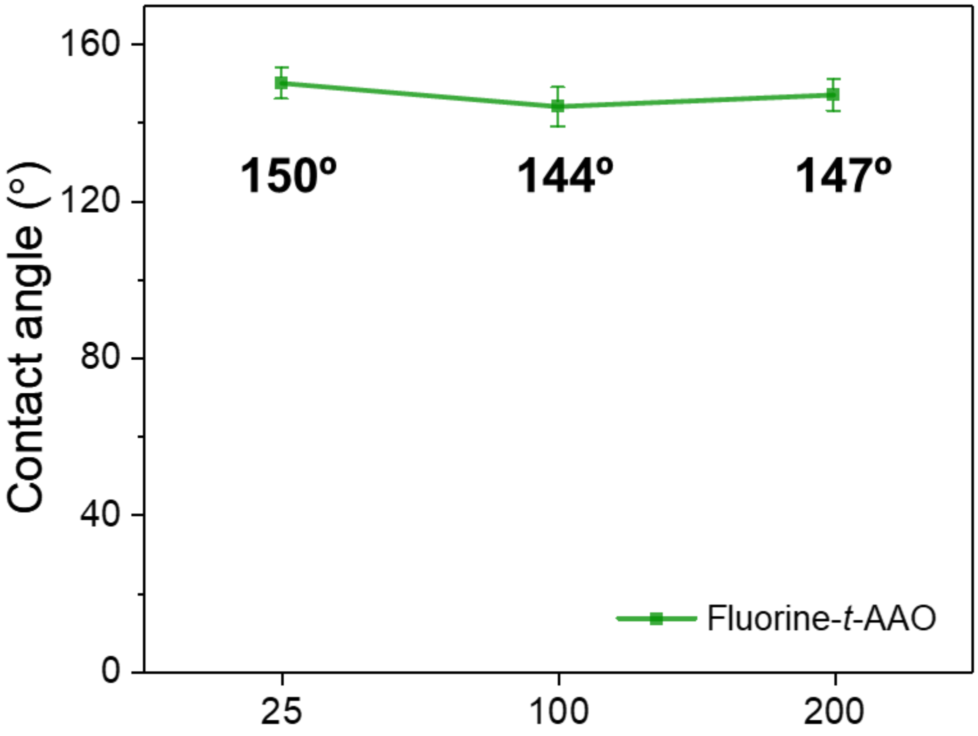

Figure S16. Stability test of an amphiphilic AAO membrane conducted by heating the membrane to different temperatures, followed by measuring the static water contact angles of the sample.

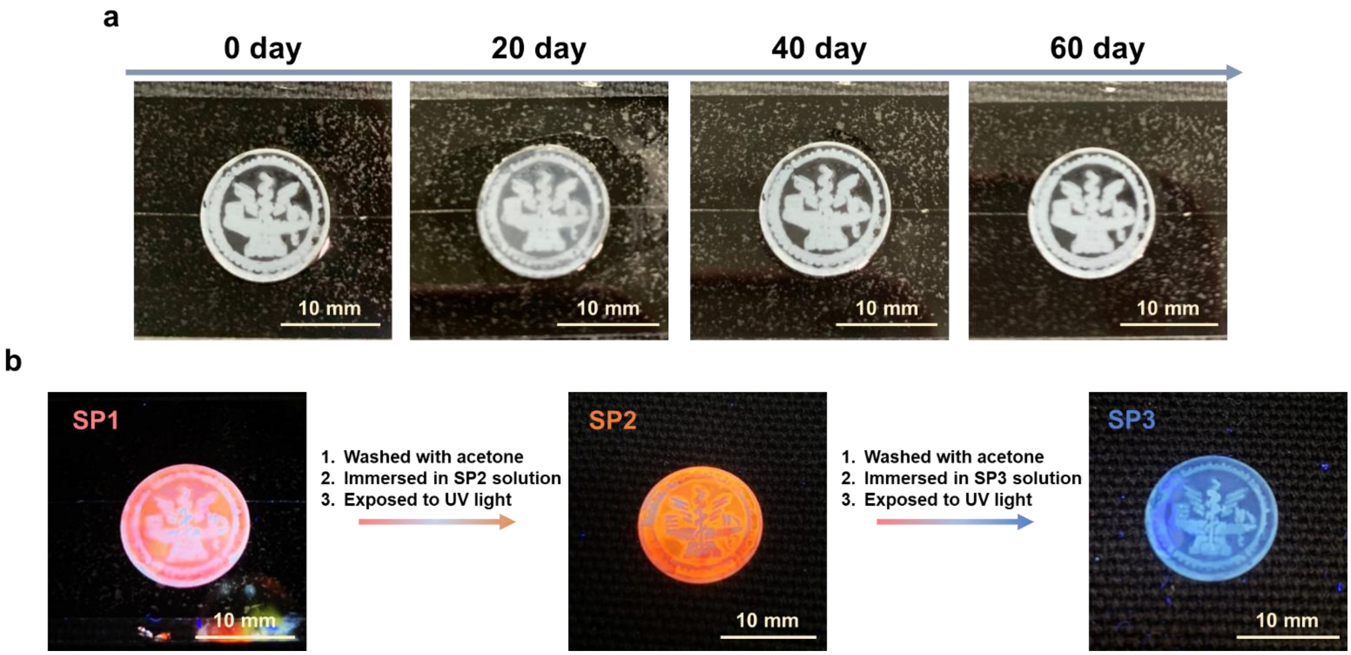

Figure S17. (a-b) Real images of (a) the durability test conducted by placing the NYCU-*p*-AAO membrane in ambient conditions for 0 to 60 days and (b) the reusability test performed by alternately immersing the NYCU-*p*-AAO membrane into different spiropyran (SP1, SP2, and SP3) solutions, exposing the membrane to 365 nm UV light, and then washing with acetone.
